# A pilot intervention to improve the management of urinary tract infections in outpatient settings

**DOI:** 10.1017/ash.2025.10228

**Published:** 2025-12-18

**Authors:** Karl J. Madaras-Kelly, Trisha Nakasone, Saba Maghari, Janice Taylor, McKenna Nevers, Jacob Crook, Benjamin Brintz, Jordan B. Braunfeld, Matthew Bidwell Goetz, Matthew Samore

**Affiliations:** 1 Coll of Pharmacy, Idaho State Univ. Meridian, ID, USA; 2 Boise Veterans Affairs Medical Center, Boise, ID, USA; 3 VA Palo Alto Healthcare System, Palo Alto, CA, USA; 4 VA Sierra Nevada Healthcare System, Reno, NV, USA; 5 School of Medicine, University of Utah, Salt Lake City, UT, USA; 6 VA Salt Lake City Healthcare System, Salt Lake City, CA, US; 7 Evergreen Health, Kirkland, WA, USA; 8 Greater Los Angeles VA Healthcare System & David Geffen School of Medicine at UCLA, Los Angeles, CA, USA

## Abstract

**Objective:**

To describe the implementation of an outpatient UTI intervention and its impact on UTI management.

**Design:**

Quasi-experimental retrospective controlled study.

**Participants:**

Outpatient clinicians practicing in emergency and primary care settings within 8 Veterans Affairs Medical Centers.

**Methods:**

An intervention conducted utilizing the CDC Core Elements Antibiotic Stewardship framework between September 2022 and July 2023. Actions included academic detailing, audit feedback, and updated reflex culture procedures. Logistic regression adjusted for covariates (risk ratio [RR], 95% confidence interval [CI]), and a difference-in-differences (DID) analysis compared multiple UTI management metrics between intervention and control facilities.

**Results:**

There were 278,419 and 157,067 genitourinary (GU) tract qualifying visits [mean (SD) age 71.7 (13.9), 92.6% male] within 8 intervention and 8 control sites, respectively. Antibiotic prescribing rates for a broad-based GU tract metric that included UTIs pre/post implementation were [*N,* (%)] 12,688 (8.0) and 4,062 (8.0) within intervention sites and 5,686 (6.3) and 1,920 (6.8) within control sites, respectively [DID aRR 0.97 (0.92, 1.02)]. Appropriate treatment selection for uncomplicated UTI (uUTI) pre/post implementation was 5,994(76.9) and 1,945(79.9), compared to 2,519(74.6) and 977(82.1) within control sites, respectively [DID aRR 0.94 (0.91, 0.98)]. uUTI appropriate treatment duration pre/post implementation was 5,709 (73.3) and 1,927 (79.2), compared to 2,469 (73.1) and 869 (73.0) within control sites, respectively [DID aRR 1.08 (1.03, 1.13)]. No evidence of diagnostic shifting or return GU visits post-implementation was observed.

**Conclusions:**

Implementation of an outpatient UTI intervention in a predominantly male population was feasible but marginally effective.

## Background

Urinary tract infection (UTI) is a common diagnosis where antibiotics are prescribed.^1–3^ Overdiagnosis and sub-optimal treatment of UTIs in outpatients is frequent, which contributes to antibiotic resistance.^4–7^ Infectious Diseases Society of America (IDSA) guidelines for uncomplicated UTI management are outdated and undergoing revision.^8^ Recently published IDSA guidelines for complicated UTI (cUTI) and other references have classified lower tract infections without signs or symptoms of systemic illness, upper tract infection, or prostatitis (in men) as uncomplicated (uUTI) in both men and women.^9–12^ These references recommend preferential selection of fluoroquinolone sparing regimens and short treatment courses for men (7 d) or less for women with uUTI.^10–12^ The Veterans Healthcare Administration (VHA) is the largest U.S. healthcare system with over 130 medical centers (VAMCs) and 3,300 outpatient clinics. A recent quality improvement analysis of outpatients with positive urine cultures (UC) indicated that approximately 40% of patients with asymptomatic bacteriuria (ASB) were prescribed antibiotics, and preferred antibiotics and treatment duration were prescribed in approximately half the remaining cases.^5^ Based on these findings, a pilot project (e.g., UTI campaign) was developed to improve outpatient UTI management. This manuscript describes the implementation of the UTI campaign and its impact on outpatient UTI management.

## Methods

Antimicrobial stewards in select VAMCs in the Western U.S. were invited to participate in the intervention by Sierra Pacific Veterans Integrated Service Network (VISN 21) administrators and study investigators. Participation required that each VAMC gain commitment from their Chief of Staff and Antimicrobial Stewardship committee to pilot an outpatient UTI management intervention.

### Intervention development

The intervention was developed based on the CDC outpatient antibiotic stewardship core elements framework.^13^ Select members of the VHA Antimicrobial Stewardship Taskforce (ASTF) developed materials for the intervention.^14^ Preparation included development of campaign key messages (Table 1) and educational materials utilized for academic detailing on UTI management. (Supplement 1a & 1b).^15^ Academic detailing consists of noncommercial, peer-to-peer communication using reinforcement techniques to facilitate change in prescribing practices.^16^ Additional campaign materials included a step-by-step implementation guide, kick-off slide set, and example UTI-specific computerized electronic order menus.

**Table 1.** Key messages of pilot UTI campaign

Diagnosis
Avoid ordering urinalyses/urine cultures in the absence of UTI symptoms
Urinalysis findings and altered mental status should not be used alone to establish a diagnosis of UTI
Assess for other potential etiologies of urinary symptoms
Differentiate between cystitis, pyelonephritis, and prostatitis; identify need for additional workup

Treatment
Only treat asymptomatic bacteriuria before invasive urological procedures or in pregnant patients
Assess patient-specific risk for antibiotic resistance and adverse events
Only prescribe fluoroquinolones in the absence of appropriate alternatives
Treat most UTIs for 7 days or less

UTI, urinary tract infection.

### Metric development and validation

Metrics were developed (Table 2) to align with key messages in support of UTI stewardship in primary care (PC) and emergency department (ED) settings.^5,17^ Diverse metrics were designed to capture UTI overdiagnosis, antibiotic selection, treatment duration, and appropriate utilization of diagnostic tests. Because limiting the analysis to UTI diagnosis codes did not fully capture use of antibiotics for suspected UTI, a broad set of genitourinary (GU)-related International Classification of Diseases, 10^th^ revision (ICD-10) codes were utilized. GU codes were classified into tiers: tier 1 (antibiotics always indicated), tier 2 (sometimes indicated), and tier 3 (rarely indicated).^1^ Altogether, 207 GU-related codes were grouped into tiers (Supplement 2). Visits with multiple codes were assigned to the lowest tier for the visit.

**Table 2.** Metrics utilized in the pilot UTI campaign

Metric	Numerator	Denominator	Comments
A. GU diagnosis w/ an antibiotic prescribed	Applicable GU visits w/ antibiotic prescribed w/ no exclusion criteria	Applicable visits w/ no exclusion criteria	**Purpose:** Global metric used to identify priority providers for academic detailing and/or audit feedback. ***Lower relative values are better***
B. GU diagnosis where antibiotics are rarely indicated w/ antibiotic prescribed	Applicable GU visits w/ antibiotic prescribed w/ no exclusion criteria	Applicable GU visits w/ no exclusion criteria	**Purpose:** To reduce unnecessary antibiotic use and detect diagnostic shifting. Includes conditions such as BPH, or symptoms-based GU codes that overlap w/ UTI symptoms where antibiotics are rarely indicated. ***Lower relative values are better***
C. ASC w/ preferred antibiotic prescribed	Cystitis or UTI-NOS diagnoses w/ no exclusion criteria where TMP/SMX, nitrofurantoin, or cephalosporins prescribed	Cystitis or UTI-NOS diagnoses w/ no exclusion criteria where an antibiotic was prescribed. Additional exclusion of T ≥99.9 F*	Preferred therapy choices are consistent w/ national and local standards. **Purpose:** Reduce unnecessary FQ therapy. ***Higher relative values are better***
D. ASC w/ preferred antibiotic duration prescribed	Cystitis or UTI-NOS diagnoses w/ no exclusion criteria where antibiotic prescribed for ≤7days	Cystitis or UTI-NOS diagnoses w/ no exclusion criteria where an antibiotic was prescribed. Additional exclusion of T ≥99.9 F*	Professional guidelines and other references recommend treating uUTI for ≤7 days **Purpose:** Reduce unnecessary antibiotic days. ***Higher relative values are better***
E. UC ordered for patients w/ antibiotic resistance risk factors	Visits w/ a UTI diagnosis where a UC was ordered and patient had ≥ 1 antibiotic resistance risk factor	Visits w/ an UTI diagnosis and patient had ≥1 antibiotic resistance risk factor	Risk factors include history of culture of UTI antibiotic-resistant organisms, UTI antibiotic prescription, or hospital/NH discharge in ≤90 days. **Purpose:** Increase UC for patients at risk of resistant infection. ***Higher relative values are better***
F. UA associated w/ antibiotic prescription	Number of UA performed with an antibiotic prescribed within a -1/ + 5 day window	All outpatient visits	Monthly rate of UA associated w antibiotic prescription. **Purpose:** Reduce unnecessary ASB treatment. ***Lower relative values are better***

Abbreviations: GU, genitourinary; W, with; BPH, benign prostatitic hyperplasia; ASC, acute simple (uncomplicated) cystitis; UTI-NOS, urinary tract infection, not otherwise specified; F, Fahrenheit; TMP/SMX, trimethoprim/sulfamethoxazole; FQ, Fluoroquinolone; UC, urine culture; UTI antibiotic resistance (resistance to FQ, nitrofurantoin, TMP/SMX or cephalosporin), NH, nursing home; UA, urinalysis; UC, urine culture; ASB, asymptomatic bacteriuria; w/, with.

a A temperature of ≥99.9F (± 1 day) was used to exclude potentially more complicated infection.

Metric A consisted of the proportion of GU visits, including all tiers, which had an antibiotic prescription filled within −1/ + 5 days surrounding the index visit. Metric B was comprised of tier 3 codes for GU conditions from Metric A. Metrics C and D were limited to visits classified as uUTI based on presence of an ICD-10 code for acute cystitis or UTI-NOS and absence of fever (temperature ≥99.9 F).^5^ The Metric C numerator included trimethoprim/sulfamethoxazole (TMP/SMX), nitrofurantoin, or an oral cephalosporin prescribed, while the denominator included all uUTI visits with antibiotics prescribed. Metric D assessed the proportion of uUTI visits with an antibiotic duration prescribed of ≤7 days.^5^ In Metric E, the denominator included all UTI visits with an antibiotic resistance risk factor present (≤90-day history of positive UC for antibiotic-resistant organisms, antibiotic prescription, or hospitalization)^5,10–11,17–20^; the numerator included all visits in the denominator with a UC collected. Metric F measured the number of urinalyses (UA) performed with an antibiotic prescribed within the (−1/ + 5 d) time window around UA collection, per 1,000 patient visits per month.

A dashboard was developed to display metrics, and a chart-level validation was conducted.^17^ Records for review were randomly selected based on strata [tier, setting (ED vs PC), and antibiotic prescription versus not]. Two reviewers (JB, KMK) independently evaluated records to determine if all inclusion/exclusion criteria were met. Reviewers were blinded to visit diagnostic codes and antibiotic prescription and used clinical judgment to assign a diagnosis and record their confidence in this diagnosis using a Likert scale (1 = highly uncertain, 5 = highly certain). Reviewers also rendered judgments regarding the appropriateness of antibiotic treatment using a Likert scale (1 = highly disapprove, 5 = highly recommend). A Likert score of ≥ 4 was considered a positive endorsement for analysis. Quantitative analysis was used to determine visit placement into tiers, relationships between visit attributes and prescription of antibiotics, and agreement in antibiotic prescription between the reviewer and the visit clinician.

### Intervention implementation

**Figure 1.**
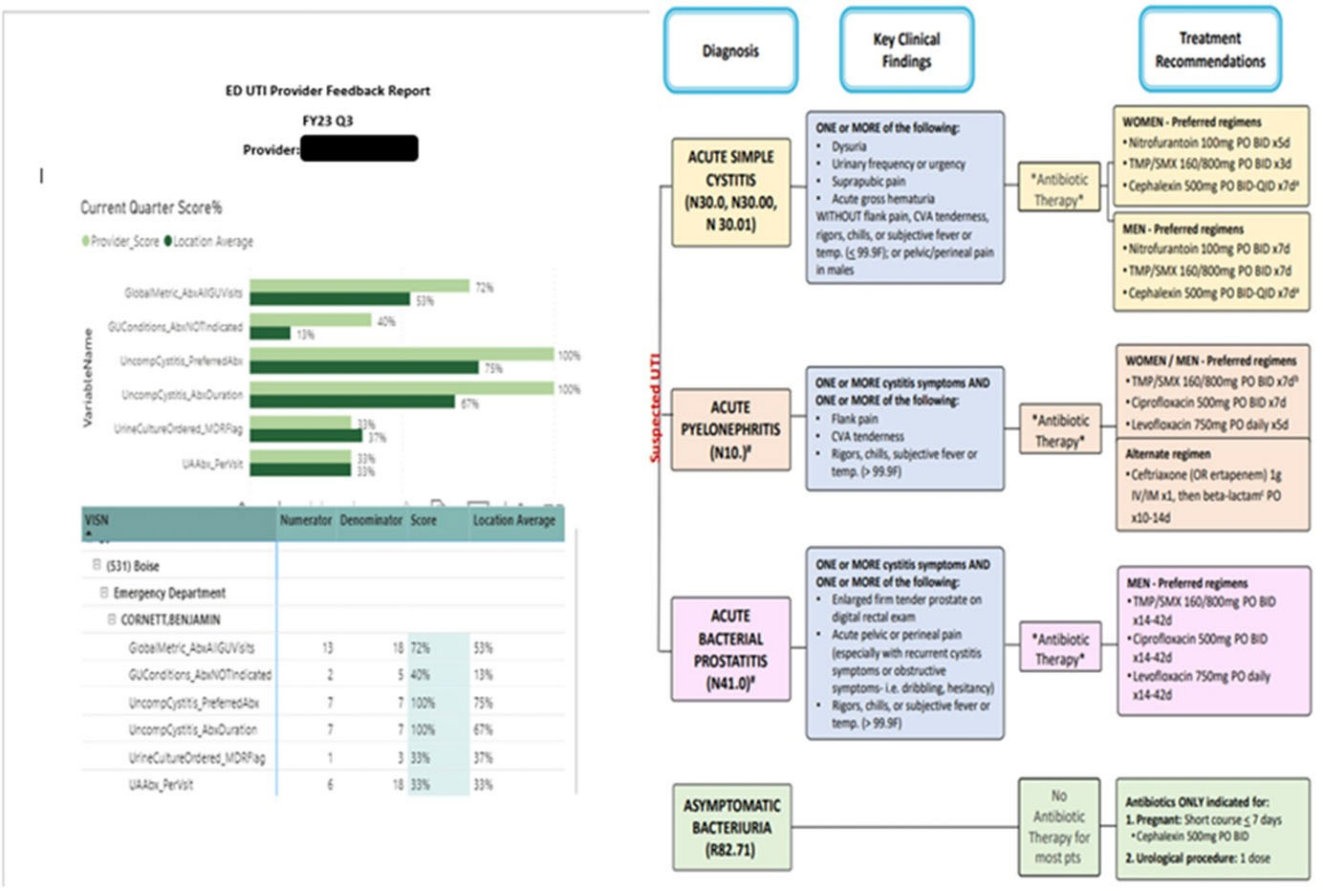
The Figure depicts a two-sided UTI campaign audit-feedback report for an ED clinician. The clinician’s performance is compared to the mean of other clinicians practicing in their ED or PC setting. Reports were emailed or printed and shared directly with clinicians. The acute uncomplicated cystitis definition utilized in the report is intended to reflect the definition for uncomplicated UTI.

The campaign was launched in fall 2022, utilizing a staggered rollout based on facility preference. Stewards were encouraged to use the implementation guide. Stewards were instructed to gain commitment through discussion with facility administration, recruit ED/PC clinical champions, and identify personnel to deliver the intervention. Next, deliver a “Kick-Off” presentation to clinicians on the purpose, key messages, and planned intervention. Facilities were instructed to update UTI electronic order pathways and reflex UC policies if not already aligned with key messages. Stewards were encouraged to track and report campaign metrics to stakeholders through a review of aggregate baseline performance and identification of “clinicians of concern” through metric review. Actions included targeting clinicians of concern with education through academic detailing on key messages and delivery of audit-feedback reports at the clinic and optionally clinician-level for individuals with sufficient visits. However, the initial dashboard was not configured to generate clinician-level reports directly. Based on feedback, the dashboard was updated to allow end users to generate individual clinician-level feedback reports (Figure 1). Stewards were encouraged to prioritize the ED where UTI density was highest. Periodic virtual learning collaboratives for stewards were implemented in January 2023, where barriers and strategies could be shared.

### Intervention sustainment

The intervention continued through June 2023, when an assessment was developed to ascertain the extent of alignment with the implementation guide, gauge intervention intensity, barriers and enablers, and suggestions for pilot refinement. The assessment, consisting of item selection, short answer, and free text response questions, was distributed to steward leads at each facility. Based on feedback, VISN 21 adopted Metrics C and D as quality improvement measures (minimum performance target of 80%) for fiscal year 2024. Stewards were encouraged to utilize the campaign materials as needed; however, the pilot was concluded.

### Cohort development and outcomes

A retrospective cohort of outpatients who met criteria for inclusion was developed for visits between 3/15/2020 and 6/30/2024 from data within the VHA Corporate Data Warehouse (CDW).^21^ Inclusion required a visit with a GU ICD-10 diagnosis code in ED or PC settings. Visits with a UTI diagnosis within <30 days, competing tier 1 or 2 coded diagnoses,^1^ hospital discharge within ≤7 days, urological procedure within ± 7 days, pregnancy, or spinal cord injury, marrow or organ transplant in the prior 2 years were excluded (Supplement 2).^5^

Data extracted included demographics, visit date, maximal temperature (± 1 d) of visit, UA/UC results, ICD-10 diagnosis, procedures, treatment date, antibiotic, treatment duration, clinician type, clinic setting, facility, antibiotic resistance risk factors, and outcomes.

The primary outcome was a change in the performance rate for each metric pre /and postintervention implementation. A secondary outcome included GU-related return visits between days 7–21 postindex visit date for uUTI pre/post implementation. Additional analyses evaluated the rate of performance on Metrics C and D during the maintenance phase and whether clinicians altered their diagnostic coding practices for uUTI relative to other select GU-diagnoses pre /post implementation.

Two intervention time-periods were evaluated: preimplementation 3/15/2020–9/30/2022 and post-implementation 01/01/2023–9/30/2023. As the intervention was initiated at slightly different time-points in facilities, data during a 3-month transition period (10/1/2022–12/31–22) was excluded. A secondary intervention analysis and time-point included preUTI campaign maintenance data from 01/01/2023–09/30/23 and postintervention maintenance data from 10/01/23–07/01/24.

### Statistical analysis

Interrupted time series analyses compared outcomes for intervention facilities with a control VISN. The control was chosen based on the average monthly Euclidean distance of metric rates before the first intervention between each VISN and VISN 21.^22^ Regression models were fit using generalized estimating equations (GEE) on each outcome with clustering by facility, assuming an exchangeable covariance matrix, or equal correlation between all subjects within a facility.^23^ All models assumed a log link to directly estimate the relative risk of the intervention by exponentiating model covariates.^24^ Each model included a fixed indicator for the intervention facilities, an indicator for the intervention period, and their interaction. All models additionally adjusted the following as fixed effects: patient age, max temperature, setting, and secular trend designated by an increasing continuous variable incremented by quarter of the year. The main result from each model was the exponentiated covariate representing the interaction between the intervention facilities and intervention period [that is the difference-in-differences (DID) relative risk]. Post hoc contrasts were made to estimate the intervention facilities’ pre to postintervention relative risk and the control VISN’s pre to postintervention relative risk. All analyses were conducted using R version 4.4.1 using the geepack and contrast packages.

The campaign was conducted as an operations activity; however, analysis activities constitute research based on VHA Policy Handbook guideline 1,058.05. This research complies with all federal guidelines and VA policies related to human subjects’ research.

## Results

**Figure 2.**
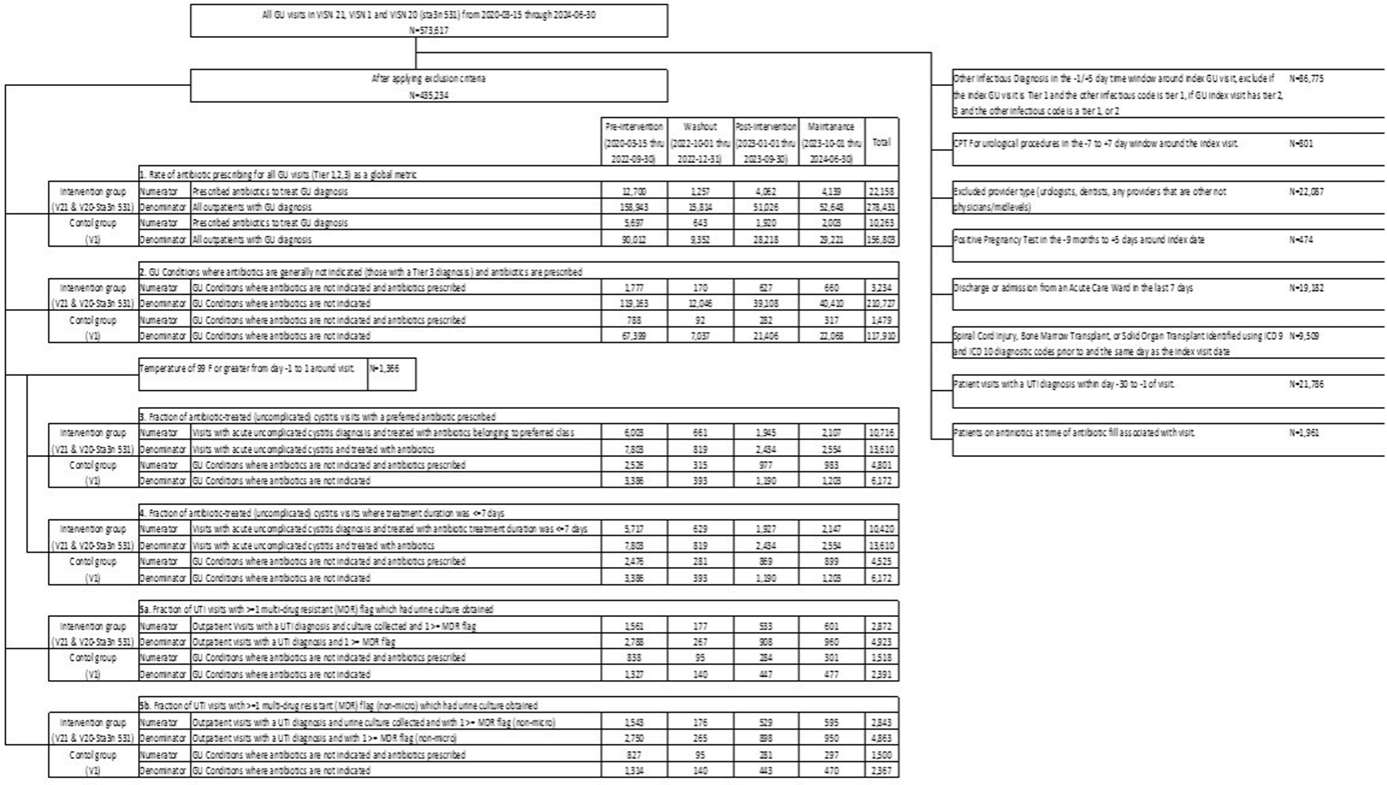
GU, genitourinary; CPT, current procedural terminology; UTI, urinary tract infection; uUTI, uncomplicated UTI; F, fahrenheit; UC, urine culture. *metric *N* values represent denominators.

A total of 573,617 visits within 16 (8 intervention, 8 control) facilities were identified, of which 435,234 (75.9%) met all criteria (Figure 2). Qualifying GU visits included tier 3 (75.0%), tier 2 (20.2%), and tier 1 (4.8%). Overall, 40,945 (9.4%) of GU visits were associated with a prescription for an oral antibiotic (Table 3); 43.4 % of prescribed antibiotics were for patients with tier 2 or tier 3 GU conditions. Most patients were elderly, male, Caucasian, afebrile, and seen in PC.

**Table 3.** Patient, clinician, and practice setting characteristics for implementation phase

	Intervention facilities (*N* = 8)		Control facilities (*N* = 8)	
Variable	PreImplementation, *N*(%)	PreImplementation, *N*(%)	PreImplementation, *N*(%)	post-implementation, *N*(%)
Patient	210,255	66,356	119,605	37,462
Mean (Median) Age (Y)	70.7 (73)	71.8 (75)	72.6 (74)	72.9 (76)
Sex (M)	193,678 (92.1)	61,106 (92.1)	112,053 (93.7)	34,910 (93.2)
**Diagnosis (Dx)**				
**Metric A (GU)**	158,943 (100)	51,026 (100)	90,012 (100)	28,218 (100)
Pyelonephritis	823 (0.4)	233 (0.4)	230 (0.2)	77 (0.2)
Prostatitis	623 (0.3)	179 (0.3)	303 (0.3)	72 (0.2)
**Metric B**	119,163 (56.7)	39,108 (58.9)	67,399 (56.4)	21,406 (57.1)
Non-UTI GU	97,239 (46.3)	32,853 (49.5)	53,023 (44.3)	17,658 (47.1)
Symptom Code	52,403 (24.9)	16,021 (24.1)	33,172 (27.7)	9,831 (26.2)
**Metric C&D (uUTI)**	7,803 (3.7)	2,434 (3.7)	3,386 (2.8)	1,190 (3.2)
**Vitals**				
Mean (±S.D.) Temp.	97.9 (0.7)	97.8 (0.7)	97.7 (0.7)	97.7 (0.7)
Temp.>= 99.9F	2,698 (1.3)	365 (0.6)	1,486 (1.2)	169 (0.5)
**Laboratory**				
UA collected	43,231 (20.6)	13,353 (20.1)	24,249 (20.3)	7,894 (21.1)
UC collected	25,331 (12.1)	7,382 (11.1)	15,285 (12.8)	4,137 (11.0)
**Provider type**				
MD	156,751 (74.6)	45,702 (68.9)	87,993 (73.6)	25,845 (69.0)
APP (NP/PA)	42,968 (20.4)	16,777 (25.3)	26,990 (22.6)	9,455 (25.2)
Other	10,536 (5.0)	3,877 (5.8)	4,622 (3.9)	2,162 (5.8)
**Treatment setting**				
ED	27,933 (13.3)	7,573 (11.4)	15,847 (13.3)	4,354 (11.6)
PC	182,322 (86.7)	58,783 (88.6)	103,758 (86.8)	33,108 (88.4)
**Antibiotic**				
Any PO antibiotic	21,664 (10.2)	6,538 (9.8)	9,746 (8.1)	3,051 (8.1)
Fluoroquinolone	3,177 (14.7)	663 (10.1)	1,647 (16.9)	362 (14.7)
TMP/SMX	3,967 (18.3)	1,090 (16.7)	1,984 (20.4)	614 (20.1)
β-lactam	9,554 (44.1)	3,008 (46.0)	3,588 (36.80)	1,202 (39.4)
Nitrofurantoin	3,316 (15.3)	1,187 (18.2)	1,593 (16,3)	544 (17.8)
Other antibiotic	1,650 (0.8)	590 (0.8)	934 (0.8)	329 (0.8)

M, male; GU, genitourinary; DX, Diagnosis based on ICD-10 code groupings; uUTI, uncomplicated UTI; Temp, Temperature; F, Fahrenheit; SD, Standard Deviation; UA, Urinalysis; UC, urine culture; MD, physician, APP, Advanced Practice Provider such as nurse practitioner (NP) or physician assistant (PA), PC/ED, primary care/emergency department (including fast track/urgent care), TMP/SMX, trimethoprim/sulfamethoxazole, PO, oral, Y, years.

### Validation

Of 618 records reviewed, 108 (17.5 %) were excluded from further review due to the presence of exclusions not documented in the CDW, which most commonly was documentation of uncoded non-GU infectious diagnoses (8.3 %). The intra-class correlation coefficient for inter-rater reliability between reviewers with respect to the recommendation for antibiotic treatment was 0.704. Projected to the source population, reviewers recommended antibiotics (mean Likert ≥ 4) in 51.3% of visits when an antibiotic was prescribed; for tier 3 visits, the reviewers agreed with the decision to prescribe antibiotics in only 18.4% of instances. Chart review and clinician documentation agreed that most visits (>85%) were associated with a diagnosis of an infection or non-infection GU condition. However, UTI or other GU infection were more commonly considered to be present based on clinician documentation (31.7%) than based on reviewer judgment (20.7%). The overall diagnosis of UTI was 132/511 (25.8%) recorded in notes by the clinician, 68/511 (13.3%) judged by the reviewer, and 94/511 (18.4%) based on clinician ICD-10 code documentation (Supplement 3).

### Pilot intervention

No significant changes in antibiotic prescribing rates were observed in intervention or control facilities for overall GU diagnoses (Metric A) or for GU conditions where antibiotics are rarely indicated (Metric B) (Table 4). Appropriate selection of antibiotics for uUTI (Metric C) increased to a greater extent in control facilities. Appropriate antibiotic duration for uUTI (Metric D) increased in intervention facilities but not control facilities. The collection of UC for patients with antibiotic resistance risk (Metric E) and the rate of UA collection with antibiotics prescribed (Metric F) were not significantly different.

**Table 4.** Pre/post implementation counts, percentages, and risk ratio (RR) + 95% CI during the implementation phase

Genitourinary urinary metrics							
	Intervention facilities			Control facilities			
Metric*	PreImplementation, *N* (%)	post-implementation, *N* (%)	aRR (±95% CI)	PreImplementation, *N* (%)	post-implementation, *N* (%)	aRR (±95% CI)	DID aRR (±95% CI)
**Metric A**	12,688 (8.0)	4,062 (8.0)	1.01(0.97,1.06)	5,686 (6.3)	1,920 (6.8)	1.04(0.99,1.1)	0.97(0.92,1.02)
**Metric B**	1,777 (1.5)	627 (1.6)	1.08(0.94,1.23)	788 (1.2)	282 (1.3)	1.11(0.93,1.31)	0.98(0.83,1.15)
**Metric C**	5,994 (76.9)	1,945 (79.9)	0.98(0.94,1.01)	2,519 (74.6)	977 (82.1)	1.04(0.99,1.08)	0.94(0.91,0.98)
**Metric D**	5,709 (73.3)	1,927 (79.2)	1.02(0.98,1.05)	2,469 (73.1)	869 (73.0)	0.94(0.90,0.99)	1.08(1.03,1.13)
**Metric E**	1,559 (57.3)	533 (59.9)	0.91(0.84,0.97)	837 (63.2)	284 (63.5)	0.86(0.79,0.94)	1.05(0.96,1.14)
Metric F**	11.2 (4.3)	12.8 (4.6)	0.86 (0.79, 0.94)	8.2 (2.8)	9.4 (2.4)	0.86 (0.8, 0.93)	1.00 (0.89–1.12)

CI: Confidence Interval; DID: Difference in Differences. Metric 6 is summarized to a monthly rate instead of a patient-level outcome.

^a^See Table 2 for description of individual metrics. aRR: adjusted relative risk.

^b^Metric F regression estimates are estimated from an unadjusted Poisson generalized estimating equation using the log number of monthly visits as an offset with clustering by facility with an exchangeable covariance matrix. Period level summaries are the average monthly rate per 1,000 visits (Metric F numerator / visits) grouped by Intervention vs Control and implementation period with monthly standard deviation in parentheses.

GU-related 21-day revisits for uUTI was low in both intervention [preimplementation (4.2%), post-implementation (4.1%); aRR 0.92 (0.68, 1.25)] and control facilities [preimplementation (3.8%), post-implementation (5.2%); aRR 1.28 (0.88, 1.85)]; [DID aRR 0.72 (0.50, 1.04)].

### post-implementation assessment

All intervention facilities completed the campaign assessment (Table 5). Facilities generally followed the implementation guide; however, while almost all facilities reported giving group-level feedback, clinician-level audit feedback occurred in less than half of the sites. Barriers included insufficient time to manage campaign activities, limited support from select ED leadership, and disagreement by some clinicians regarding rationale and evidence for uUTI in men. Enablers included automation of clinician audit-feedback report generation, detailing support materials, and select strong ED Champion support. Personnel expressed that the pilot was complex and had too many metrics to emphasize. Stewards in 7/8 (87.5%) facilities focused on improvement of treatment selection and duration for uUTI, rather than other metrics they felt were less actionable. Notable quotes included “Generally easier to impact what is prescribed and how long to prescribe than when to prescribe,” or “it’s not my role to tell a clinician he is bad in comparison to his peers….” Finally, 5/8 (62.5%) of facilities indicated they would continue to use the UTI dashboard if it were maintained.

**Table 5.** Summary of UTI campaign implementation phase assessment

Intervention element	Number (%) of intervention facilities	Comment
Utilized suggest approach protocol	8/8 (100)	Variable implementation: only two facilities implemented all elements
Held formal kick-off	7/8 (87.5)	Predominantly with ED staff but select sites did PC/facility wide
Performed academic detailing	6/8 (75.0)	Most detailing done by AD personnel, some stewards requested training
Periodically provided group level feedback to clinics	6/8 (75.0)	An additional facility provided baseline but not follow-up feedback
Provided audit-feedback to individual clinicians	3/8 (37.5)	Automated reports not available until late in intervention period.
Revised order sets/ reflex UC policies	5/8 (62.5)	Some facilities already had tools consistent with campaign key messages
**Barriers**		
Time to implement outpatient stewardship	–	Inpatient stewardship was priority and COVID-19 pandemic was waning
Tepid support from some ED leadership/clinicians	–	Select ED Chiefs limited intervention, disagreement on rationale for ASC in males
Campaign too complex	–	Too many metrics to focus. 7/8 sites focused on uUTI metrics C&D
**Enablers**		
Development of automated feedback reports	–	Customizable reports w/ peer comparison for metrics of choice for print/e-mail
Detailed and profession campaign materials	–	Especially academic detailing materials/implementation/treatment algorithms
Select strong ED Chief/Champion support	–	In select cases physicians participated in key actions enhancing acceptance

### Maintenance phase

VISN 21 adopted uUTI antibiotic selection and treatment duration metrics (Metrics C & D) as outpatient stewardship measures for FY 2024. Appropriate antibiotic selection was similar (82.1% to 81.7%) during the maintenance phase in intervention facilities and increased slightly (80.0% to 82.3%) in control facilities, [DID aRR (0.97 (0.93, 1.02)]. Appropriate antibiotic duration increased in both intervention (73.0% to 74.7%) and control facilities (79.3% to 84.6%), [DID aRR 0.96 (0.91, 1.02)].

Given the threshold of minimum performance (80%) for Metrics C and D, we anticipated diagnostic shifting by using alternative diagnostic codes included in Metrics A and B. However, no evidence of diagnostic shifting was identified (Supplement 4).

## Discussion

Analysis of this intervention revealed minimal improvement in outpatient UTI management. While slight improvements in antibiotic selection and treatment duration were observed, there was no reduction in antibiotic prescribing for GU-related conditions, including UTIs. The pilot was embedded within the Core Elements framework, allowing adoption of an implementation strategy that was patterned after a more effective prior intervention on acute respiratory tract infections within the VHA.^22,25^

The post-implementation assessment provided insight into challenges and plausible explanations for the minimal effect. The intervention key messages were broad, addressing both diagnosis and treatment as an intent of the pilot was to identify potential foci for a future VA wide intervention. A more focused intervention might have been more efficiently implemented. The intervention was initiated late in the COVID-19 pandemic, which led to delays in dashboard development, impeding timely delivery of clinician-level audit feedback. The intervention was aligned with contemporary recommendations for uUTI management, including a reduction in unnecessary prescribing of fluoroquinolones and shortened treatment duration. However, clinical practice with respect to these targets had already improved over the past decade within the VHA. These trends may have contributed to achieving and measuring further improvements. Pharmacists were predominantly responsible for delivering actions on when to prescribe antibiotics. Some stewards were not comfortable delivering key messages on diagnoses and when to prescribe antibiotics, indicating a potential intervention-personnel mismatch. This highlights the necessity for involvement of prescribing peers and leadership in delivering audit feedback and academic detailing.^16,26^ The diversity of opinion on optimal treatment for male UTIs led to mixed acceptance of select key messages. The lack of consensus of uUTI management in males is likely to be mitigated by the recently published IDSA guidelines for complicated UTI, which suggest that infection limited to the bladder can be treated with 7 days of therapy in both sexes. ^12,27,28^

Six metrics for outpatient UTI management were developed to support the pilot intervention. These metrics were designed to be applicable for feedback aggregated at the facility or clinician level and to be used to support individual case review.^5^ The development of a broad-based GU metric that included codes for UTI and other GU diagnoses with overlapping symptoms increased yield of visits where the clinician intent was to treat UTI. Chart review validated the use of a tiered approach to classify GU diagnosis codes and contributed further evidence of unnecessary antibiotic use for suspected UTI. Utilization of ICD-10 codes to assess UTI management was challenging due to complicating conditions, overdiagnosis, under-coding, and miscoding. Use of a narrower set of diagnosis codes might have reduced complexity, but at the risk of increasing susceptibility to diagnostic shifting.^22^ While diagnostic shifting was not observed, the combination of metrics A-D theoretically could capture these changes. Finally, ICD-10 39.0 (UTI-NOS) was utilized in 3/4^ths^ of visits coded for UTI. Chart validation indicated that some of these cases likely had ASB, and while patients with documented fever were excluded, some patients likely had complicated infection. While steps to address under-coding and miscoding were taken, poor coding documentation was a limitation.

Most outpatient studies have focused on diagnostic stewardship to reduce ASB treatment or bundled antibiotic stewardship interventions targeting reduced fluoroquinolone use, increased utilization of guideline recommended antibiotics, or excessive treatment duration.^29^ Diagnostic interventions have included changes in laboratory reflex UC criteria, order menus, nudges, and audit-feedback, whereas studies evaluating UTI treatment have utilized a variety of interventional techniques including academic detailing and audit-feedback.^29,30^ Diagnostic studies have primarily demonstrated decreased UC ordering or treatment of ASB, rather than reduced overall antibiotic prescribing or improved patient outcomes. In parallel to our findings, UTI treatment study results have been mixed, demonstrating improved guideline concordance with selection and duration of antibiotics but not reductions in overall antibiotic prescribing. Notably, most studies have been conducted in women, and few have been powered to assess clinical outcome. Finally, the accuracy of administrative coding for outpatient infectious conditions in non-VHA ED settings is similar to that observed in this study.^33^

Future UTI interventions should reduce the number of metrics and key messages. Metrics should align with key messages and target practice behavior in select clinical populations. The most desirable practice pattern would be to reduce clinician antibiotic prescribing for non-infectious conditions; however, if pharmacists deliver the intervention, they may be less likely to be effective at reducing total antibiotic prescribing for UTI. Actions targeting diagnostic stewardship, including appropriate ordering of urinary diagnostic tests, broadened differential for GU conditions, and accurate administrative coding, may require a different messenger (i.e., physicians, mandates, pay for performance) and systems tools (i.e,, augmented intelligence, coding decision support).

In conclusion, implementation of an outpatient UTI intervention in a predominantly male population was challenging to implement and minimally effective at decreasing overall GU antibiotic prescription.

## Supporting information

10.1017/ash.2025.10228.sm001 Madaras-Kelly et al. supplementary material 1 Madaras-Kelly et al. supplementary material

10.1017/ash.2025.10228.sm002 Madaras-Kelly et al. supplementary material 2 Madaras-Kelly et al. supplementary material

10.1017/ash.2025.10228.sm003 Madaras-Kelly et al. supplementary material 3 Madaras-Kelly et al. supplementary material

10.1017/ash.2025.10228.sm004 Madaras-Kelly et al. supplementary material 4 Madaras-Kelly et al. supplementary material

10.1017/ash.2025.10228.sm005 Madaras-Kelly et al. supplementary material 5 Madaras-Kelly et al. supplementary material

## Data Availability

The data that support the findings of this study are owned by the U.S. Department of Veterans Affairs. Restrictions apply to the availability of these data, which were used under license for the current study, and so they are not publicly available. Some data utilized in this study may be available to VA credentialed researchers upon reasonable request from the authors and with permission of U.S. Department of Veterans Affairs. In an effort to aid in transparency and foster reproducibility of research, the manuscript supplementary files include diagnostic and procedure codes and procedures utilized to develop the cohort.

## Supplementary material

The supplementary material for this article can be found at https://doi.org/10.1017/ash.2025.10228.
