# Supplementary material for: A pilot intervention to improve the management of urinary tract infections in outpatient settings
Source: Antimicrob Steward Healthc Epidemiol. 2025 Dec 18;5(1):e338. doi: 10.1017/ash.2025.10228 (PMC12722549; doi:10.1017/ash.2025.10228)
Supplement: Madaras-Kelly et al. supplementary material 1 — Madaras-Kelly et al. supplementary material [file S2732494X25102283sup001.pdf]

Outpatient Management of Urinary Tract Infections (UTI)

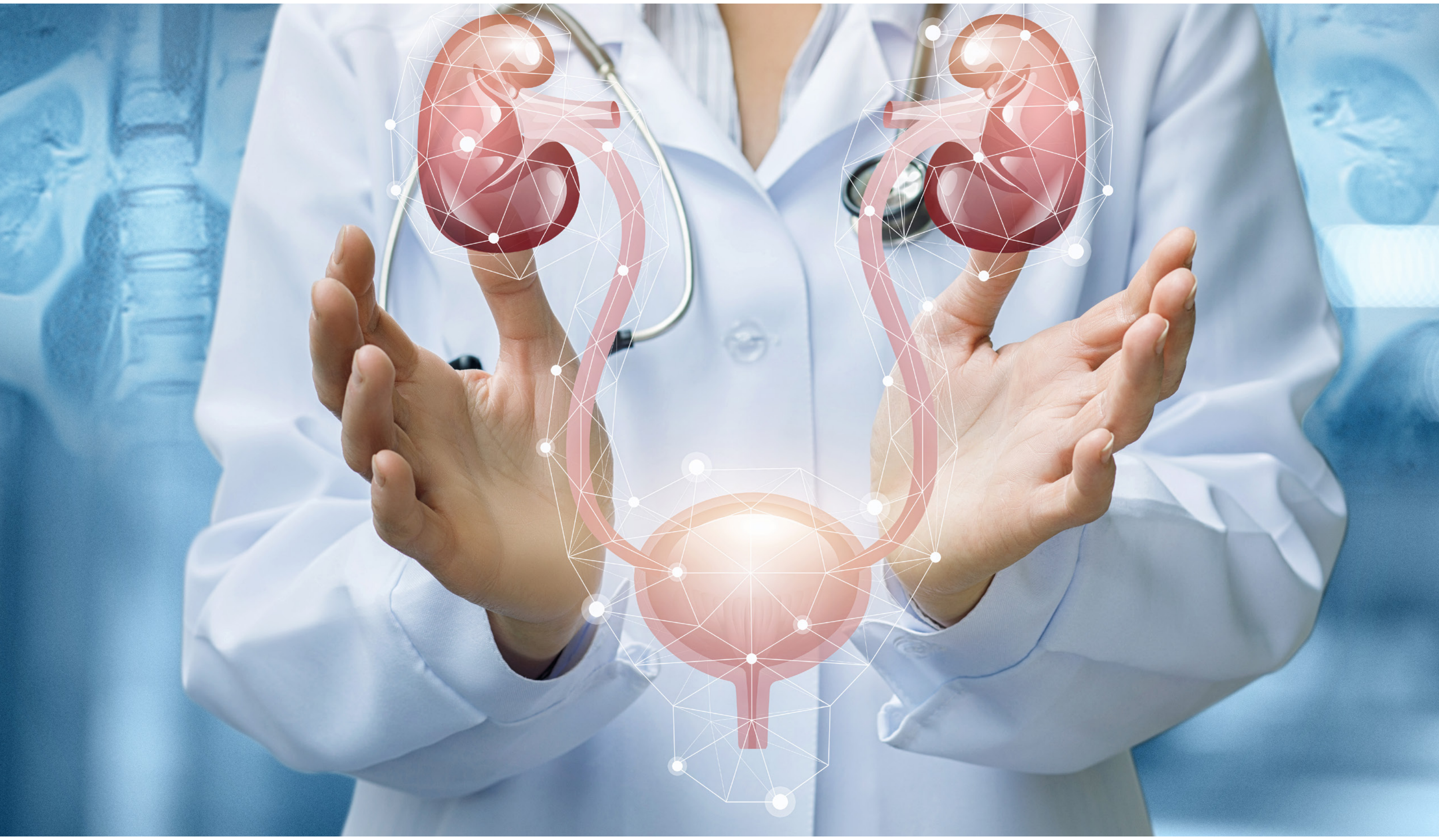

A VA Clinician's Guide to Identification and Management of UTI and Similar Clinical Conditions: Making the Correct Diagnosis and Prescribing the Correct Treatment.

VA

U.S. Department of Veterans Affairs

Veterans Health Administration

PBM Academic Detailing Services

Outpatient Management of Urinary Tract Infections (UTI)

Identification and Management of UTI and Similar Clinical Conditions: Making the Correct Diagnosis and Prescribing the Correct Treatment.

A VA Clinician's Guide

VA | U.S. Department of Veterans Affairs
Veterans Health Administration
PBM Academic Detailing Services

VISN 21 Antimicrobial Stewardship Collaborative

VA Antimicrobial Stewardship Task-force

VA PBM Academic Detailing Service

Real Provider Resources

Real Patient Results

Your Partner in Enhancing Veteran Health Outcomes

Attention Healthcare Provider:

These recommendations are intended for the diagnosis and management of urinary tract infections in immunocompetent patients. Individual patient-specific characteristics should be considered when determining appropriate therapy

VISN21 Antimicrobial Stewardship Email Group and or ASTF Contact info:
VISN21ANTIMICROBIALSTEWARDSHIP@va.gov

VA ASTF SharePoint Site:
https://dvagov.sharepoint.com/sites/VHAPBM/ASTF/SitePages/ASTF.aspx

VA PBM Academic Detailing Service Email Group
PharmacyAcademicDetailingProgram@va.gov

VA PBM Academic Detailing Service SharePoint Site
https://dvagov.sharepoint.com/sites/vhaacademicdetailing/ClassicMigration/SitePages/Home.aspx

Trends and Impact of Antimicrobial Resistance on Older Inpatients With Urinary Tract Infections (UTIs)

Urinary tract infections (UTIs) result in 8 million outpatient visits and 100,000 hospitalizations annually in the United States.[1]

Hospitalizations of elderly patients with antibiotic resistant UTIs have increased (See figure).[2,3]

Discordant empirical therapy (selection of the wrong antibiotic prior to culture & susceptibility result) is common and associated with increased clinical failure.[4]

Major drivers of antibiotic resistance include misdiagnosis of UTI, overtreatment of asymptomatic bacteriuria, sub-optimal selection and duration of antibiotic therapy.[5]

The U.S. Department of Health and Human Services established an objective for Healthy People 2030 to reduce UTI-related hospitalizations for older adults.[6]

This educational guide provides key actions that clinicians can take to improve the diagnosis and management of UTIs for veterans.

Trends in Primary UTIs: Admission and Antimicrobial Resistance From 2009–2016[2]

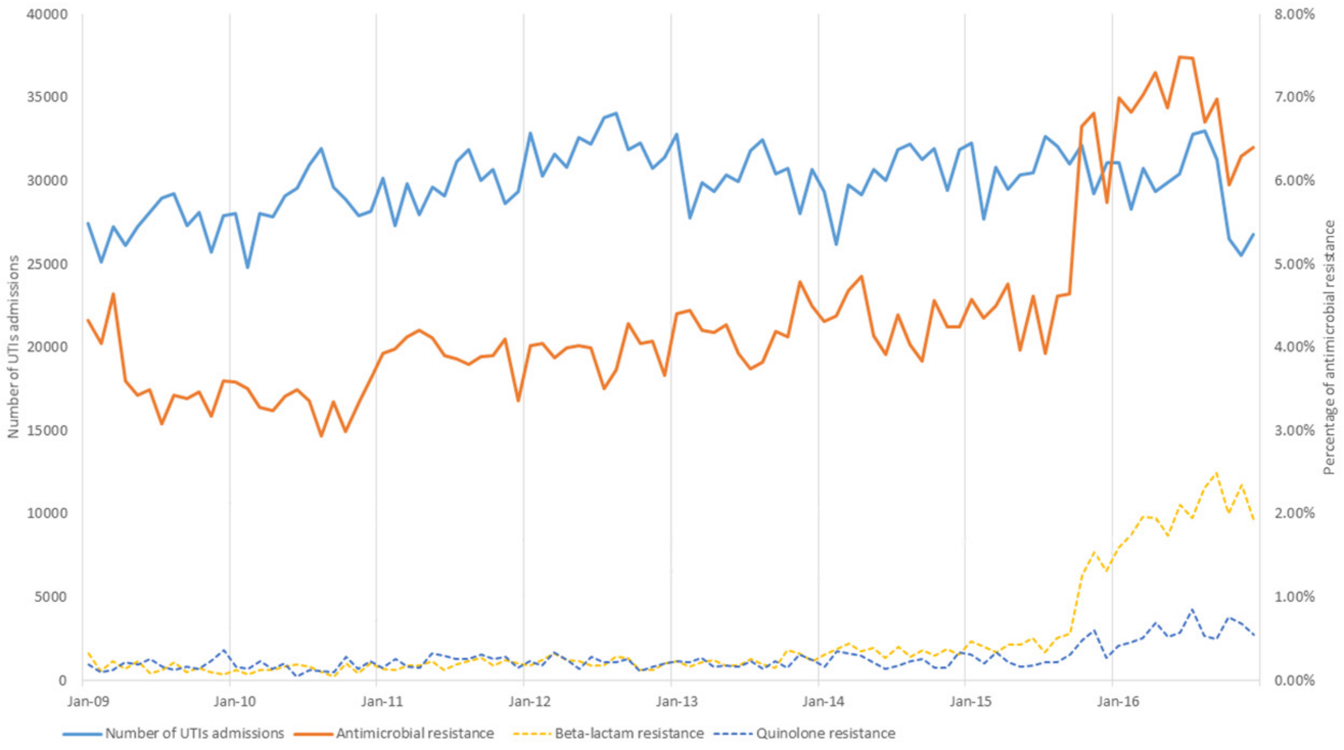

KEY MESSAGES

Diagnostics

- Avoid ordering urine cultures in the absence of UTI symptoms.
- Urinalysis findings and altered mental status should not be used alone to establish a diagnosis of UTI.
- Assess for other potential etiologies of urinary symptoms.
- Differentiate between cystitis, pyelonephritis, and prostatitis; identify need for additional workup.

Treatment

- Only treat asymptomatic bacteriuria before invasive urological procedures or in pregnant patients.
- Assess patient-specific risk for antibiotic resistance and adverse events.
- Only prescribe fluoroquinolones in the absence of appropriate alternatives.
- Treat most UTIs for 7 days or less.

Asymptomatic Bacteriuria (ASB) Refers to the Presence of Bacteria in the Urine Without Symptoms of Infection

In a recent VA MEDSAFE utilization review in 31 VAMCs, 50% of 3,255 positive urine cultures were collected in veterans with no documented UTI symptoms.[7]

Routine ordering of urinalysis (UA) or reflex of urine specimens for culture without a clear indication is unnecessary and can lead to overtreatment of UTI.

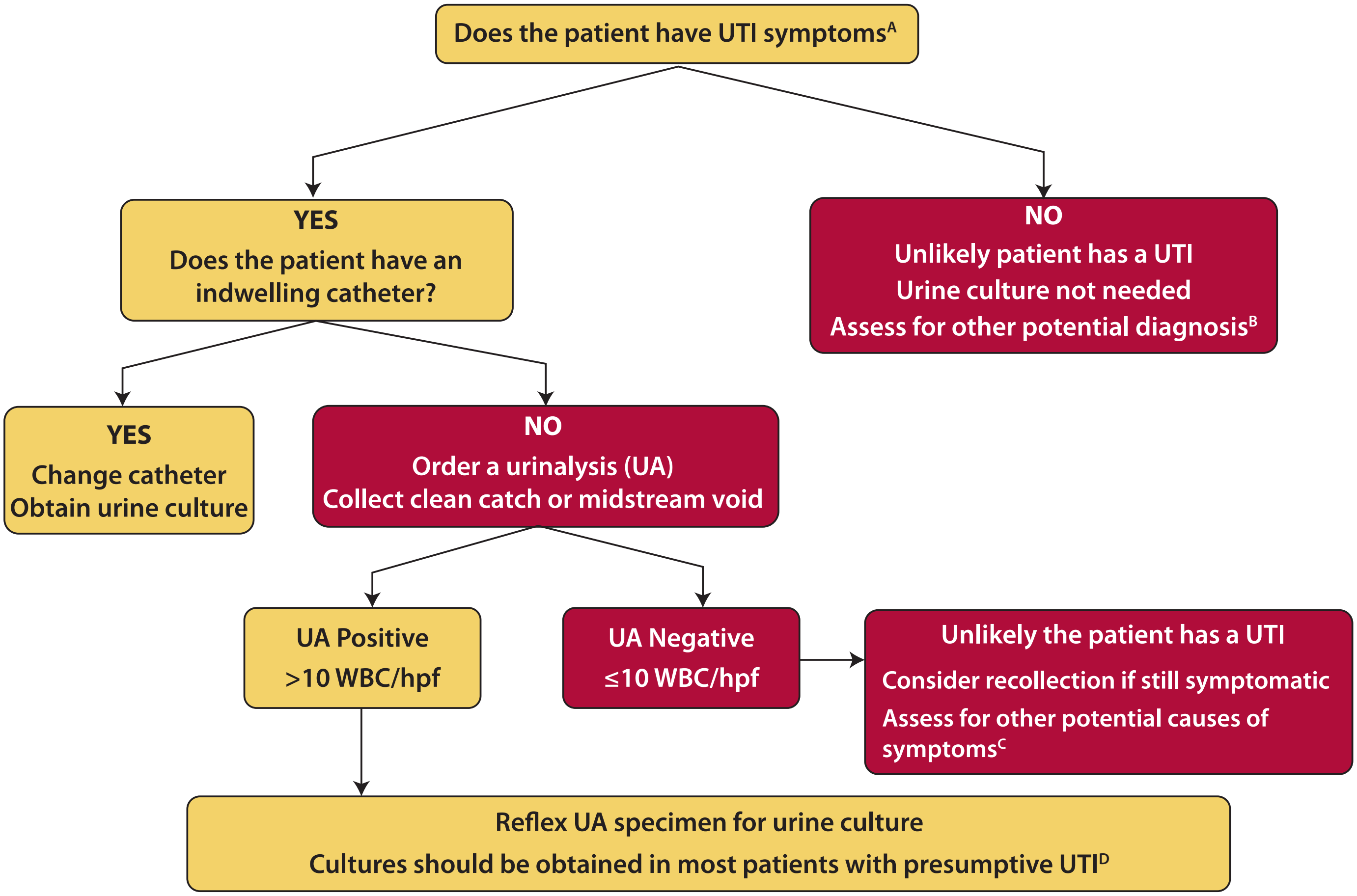

A - See page 8 for list of symptoms, B - See page 7 for diagnostic differential, C - See page 6 for additional UA interpretation, D - Nonpregnant women of child-bearing age without risk factors for antimicrobial-resistant UTI do not necessarily need urine cultures. See page 10 regarding pregnancy and page 11 for a list of common antibiotic resistance risk factors.

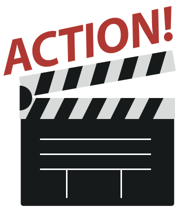

- Assess patient UTI symptoms before ordering urine cultures and only order UAs and urine cultures when clinically necessary.
- When reviewing reflex culture results, interpret within the context of specific UTI symptoms.

Urinalysis Findings and Altered Mental Status Should Not be Used Alone to Establish a Diagnosis of UTI

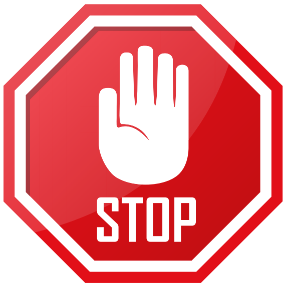

UTI is not a laboratory - defined diagnosis!

Mental status changes and falls can have many causes!

Diagnosis should be based on UTI-specific symptoms AND laboratory findings

? Did You Know

UA is among the most commonly ordered laboratory tests in the VA.

While select UA components may screen for UTI, if interpreted in isolation, they lack sensitivity and specificity for UTI.

Altered mental status changes in the elderly without localized UTI symptoms or systemic signs of infection have poor specificity for UTI diagnosis.[4,8]

Common Misperceptions Leading to the Assumption the Patient has a UTI[8]	
**The UA is cloudy or smells bad**	• Urine color, clarity, or odor should not be used alone to diagnose a UTI.<br>• Visual inspection of urine has poor sensitivity to detect UTI and foul odor is dependent upon hydration status and urea concentration.
**The UA is positive for leukocyte esterase or pyuria**	• Positive leukocyte esterase or pyuria should not be used alone to diagnose UTI.<br>• Degree of pyuria does not define a UTI.<br>• Moderate pyuria has many causes: renal failure, catheterization, cancer, and UTI.<br>• UTI symptoms in the absence of pyuria suggest an alternative diagnosis.
**The UA is positive for nitrates**	• Positive nitrates only indicate that certain bacteria are present in the urine and should not be used alone to support a diagnosis of UTI.<br>• Absence of nitrates AND pyuria generally rules out UTI; search for alternative diagnosis if symptomatic.
**The UA has bacteria present which will progress to a UTI**	• Bacteriuria is a common finding in the elderly and increases with age.<br>• The incidence of bacteriuria associated with indwelling catheterization is 3%–8% per day.<br>• Untreated asymptomatic bacteriuria is NOT associated with development of complications such as UTI or sepsis.
**Falls or acute mental status changes are usually caused by UTI**	• Falls or acute mental status changes in the elderly can be caused by deconditioning, dehydration, hypoxia, medications, or infection including UTI.<br>• Evidence of systemic infection such as fever/leukocytosis or localized symptoms of UTI should be present to diagnose UTI in patients with acute mental status changes.

Assess for Other Potential Etiologies of Urinary Symptoms

? Did You Know

Many diagnoses can present with signs or symptoms that overlap with UTI.

Delay in making the correct diagnosis can result in worse outcomes (e.g., bladder cancer).

Failure to recognize complicating conditions of UTI results in clinical failure or recurrent infection.

Chronic or slowly worsening urinary frequency and urgency in male patients without perineal/pelvic pain, or systemic signs of infection may indicate benign prostatic hypertrophy (BPH), not infection.

Symptom	Potential common non-infectious causes of symptoms often attributed to UTI
**Urinary frequency/ urgency**	Benign prostatic hypertrophy, urethral stricture, overactive bladder, stress incontinence, overflow incontinence, excessive fluid/caffeine intake, diuretics, hyperglycemia
**Dysuria**	Urethral stricture, urothelial malignancy, sexually transmitted infection, urinary spasm, Candida vaginitis
**Suprapubic pain/ pelvic discomfort**	Urinary obstruction, constipation, diverticulitis, genitourinary malignancy, sexually transmitted infection, catheter misplacement, intra-abdominal etiology such as diverticulitis or appendicitis
**Flank pain**	Renal infarct, malignancy, pleural pathology, nephrolithiasis
**Testicular pain or tenderness**	Epididymitis (unilateral pain), spermatic or testicular torsion
**Hematuria**	Urothelial/renal malignancy, nephrolithiasis, trauma, acute kidney injury, sexually transmitted infection, excessive anticoagulant therapy, tuberculosis, sickle cell disease, chronic NSAID use
**Altered mental status**	Medication interactions, sundowning (especially with change in care setting), poor oral intake, electrolyte disturbance, trauma/head injury, stroke, CNS space-occupying lesion, infection elsewhere

Pyelonephritis Symptoms

≥1 Cystitis symptom AND ≥1 of the following:

- Flank pain
- CVA tenderness
- Rigors
- Temp > 99.9F
- 1 or more SIRS* criteria

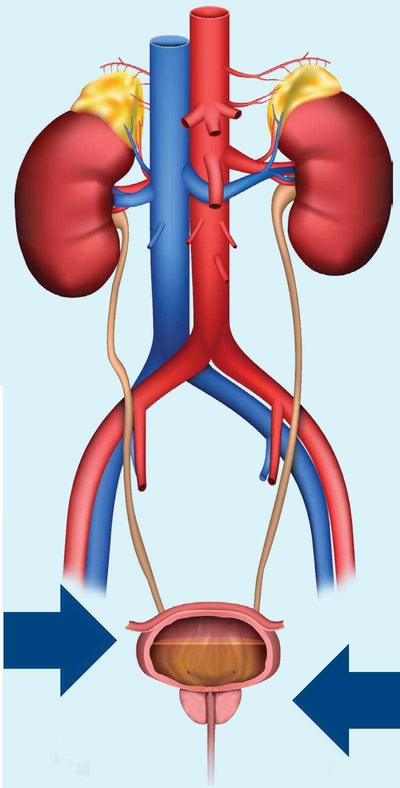

Cystitis Symptoms

≥1 of the following:

- Dysuria
- Frequency
- Urgency
- Suprapubic pain
- Acute gross hematuria

Prostatitis Symptoms

≥1 Cystitis symptom AND
≥1 of the following:

- Pelvic OR perineal pain
- Obstructive symptoms (dribbling, hesitancy)
- Temp > 99.9F
- 1 or more SIRS* criteria

*SIRS=The Systemic Inflammatory Response Syndrome (SIRS) is the occurrence of at least two of the following criteria: fever >100.4F or hypothermia <96.8F, tachycardia >90 beats/min, tachypnea >20 breaths/min, leukocytosis >12x109/L or leukopenia <4*109/L

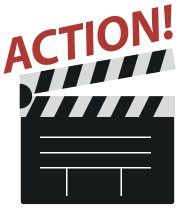

- Obtain an accurate prior medical history, review of symptoms, and physical examination.
- Consider alternative diagnoses in your differential, especially if the findings are not entirely consistent with UTI.

Differentiate Between Cystitis, Pyelonephritis, and Prostatitis; Identify Need for Additional Workup

? Did You Know

UTIs can occur in different anatomical locations in the genitourinary tract.

Common UTI diagnoses include cystitis, pyelonephritis, and prostatitis (see table below)

The diagnosis and management considerations are dependent upon anatomical differences between sexes.

Response to antibiotic therapy and treatment duration are diagnosis dependent.

Diagnosis[9-12]	Signs/Symptoms
	**Women**
**Acute Simple Cystitis**	**ONE or MORE of the following:**<br>■ Dysuria<br>■ Urinary frequency or urgency<br>■ Suprapubic pain<br>■ Acute gross hematuria<br>**WITHOUT** flank pain, CVA tenderness, rigors, chills, or subjective fever or temp. (<99.9F)
**Acute Pyelonephritis**	**ONE or MORE cystitis symptom WITH at least ONE of the following:**<br>■ Flank pain<br>■ CVA tenderness<br>■ Rigors, chills, subjective fever or temp. (≥99.9F)<br>■ OR 1 or more SIRS criteria

Presence of complicating conditions can increase the risk for recurrent infection or failing therapy. Complicated cases, patients with recurrent symptoms, or antibiotic resistant infections such as extended spectrum beta-lactamase producing organisms (ESBLs) may require referral to urology or infectious diseases (ID). Other reasons for urology referral include history of bladder cancer; history or risk of urethral strictures, underlying neurologic disease that could be associated with a neurogenic bladder; persistent or recurrent urinary retention (PVR >150 mL in older adults). **Urgent referral** is indicated in the settings of renal compromise due to urinary retention, palpable bladder on exam or unexplained hematuria.

Diagnosis	Signs/Symptoms
	**Men**
Acute Simple Cystitis	**ONE or MORE of the following:**<br>■ Dysuria<br>■ Urinary frequency or urgency<br>■ Suprapubic pain<br>■ Acute gross hematuria<br>**WITHOUT** the following: flank pain, CVA tenderness, pelvic or perineal pain, rigors, chills, or subjective fever or temp. (<99.9F)
Acute Pyelonephritis	**ONE or MORE cystitis symptom WITH at least ONE of the following:**<br>■ Flank pain<br>■ CVA tenderness<br>■ Rigors, chills, subjective fever or temp. (≥99.9F)<br>■ OR 1 or more SIRS criteria
Acute Bacterial Prostatitis	**ONE or MORE cystitis symptom WITH at least ONE of the following:**<br>■ Enlarged firm tender prostate on digital rectal exam<br>■ Acute and severe pelvic or perineal pain (especially with recurrent cystitis symptoms or obstructive symptoms- i.e. dribbling, hesitancy)<br>■ Rigors, chills, or subjective fever or temp. (≥99.9F)<br>■ OR 1 or more SIRS criteria

Presence of complicating conditions can increase the risk for recurrent infection or failing therapy. Complicated cases, patients with recurrent symptoms, or antibiotic resistant infections such as extended spectrum beta-lactamase producing organisms (ESBLs) may require referral to urology or infectious diseases (ID). Other reasons for urology referral include: history of prostate or bladder cancer; history or risk of urethral strictures, underlying neurologic disease that could be associated with a neurogenic bladder; persistent or recurrent urinary retention (PVR >150 mL in older adults). **Urgent referral** is indicated in the settings of renal compromise due to urinary retention, palpable bladder on exam or unexplained hematuria, or abnormal digital rectal exam findings that are suspicious for prostate cancer.

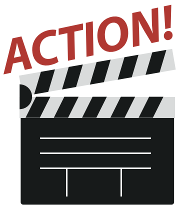

- Classify sex-specific UTI signs and symptoms and identify the anatomical location of infection when possible.

Presence of complicating conditions can increase the risk for recurrent infection or failing therapy.

Complicated cases, patients with recurrent symptoms, or antibiotic resistant infections may require referral to urology or infectious disease

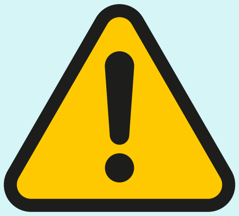

Any of the following can result in a complicated UTI

- Indwelling urinary catheter
- Residual urine (>150 ml) after voiding (neurogenic bladder, benign prostatic hypertrophy)
- Obstructive uropathy (nephrolithiasis, stricture, fibrosis)
- Azotemia caused by intrinsic renal disease

Examples of patients that may require referral:

- History of recurrent UTIs (>3 per year), particularly those caused by multi-drug resistant bacteria including extended spectrum beta-lactamase producing organisms (ESBLs)
- Pregnant women
- History of prostate or bladder cancer
- History or risk of urethral strictures
- Underlying neurologic disease that could be associated with a neurogenic bladder
- Persistent or recurrent urinary retention (PVR >150 mL in older adults)

URGENT REFERRAL

- **Renal compromise due to urinary retention**
- **A palpable bladder on exam or unexplained hematuria**
- **Abnormal digital rectal exam findings that are suspicious for prostate cancer**

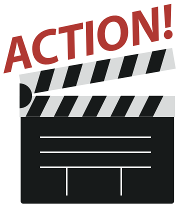

- Identify risk factors for complicated infection and seek expert advice and timely referral for complicated cases.

Only treat asymptomatic bacteriuria before invasive urological procedures or in pregnant patients.

? Did You Know

A systematic review evaluating antibiotic treatment of ASB identified an increased risk in antibiotic adverse events (RR 3.77; 95% CI, 1.40, 10.15).[13]

The treatment of ASB did NOT decrease the rate of symptomatic UTIs, infectious complications, or deaths.

Treatment of ASB is associated with subsequent development of antimicrobial resistant UTIs.

Outcomes	Illustrative comparative risks (95% CI)		Reflective effect (95%CI)	# of Participants (Studies)	Quality of the evidence (GRADE)
	**NO TREATMENT** (Assumed risk)	**ANTIBIOTICS** (Corresponding risk)			
**Number of subjects who developed symptomatic UTI** (6 months to 1 year)	**Medium risk population**		RR 1.11 (0.51 to 2.43)	1046 (5)	♦♦♦♦ moderate
	200 per 1000	222 per 1000 (102 to 486)			
**Number of subjects who developed complications** (10 months to 3 years)	**Medium risk population**		RR 0.80 (0.36 to 1.75)	814 (3)	♦♦♦♦ moderate
	30 per 1000	24 per 1000 (11 to 52)			
**Death** (6 months to 8years)	**Medium risk population**		RR 0.99 (0.70 to 1.41)	761 (6)	♦♦♦♦ moderate
	140 per 1000	138 per 1000 (98 to 197)			
**Number of subjects who develop any adverse event** (42 days to 10 months)	**Medium risk population**		RR 3.77 (1.40 to 10.15)	248 (3)	♦♦♦♦ moderate
	40 per 1000	151 per 1000 (56 to 406)			
**Number of subjects with bacteriological care** (42 days to 4 years)	**Medium risk population**		RR 2.32 (1.11 to 4.83)	1154 (9)	♦♦♦♦ moderate
	430 per 1000	997 per 1000 (477 to 2077)			

Antibiotic Treatment of ASB is Only Appropriate in Pregnancy and Prior to Select Invasive Urological Procedures

Pregnancy	Invasive Urological Procedures
Screen at 12–16 weeks gestation or first visit (consider rescreening high-risk women)<br><br>Treat ≥10^5 CFU/ml based on susceptibility results<br><br>Short courses (≤7 d) of β-lactams are preferred<br><br>Repeat culture and retreat if necessary	Single dose prophylaxis where significant mucosal bleeding is indicated including but not limited to:<br><br>Transurethral resection of prostate (TURP), prostate biopsy, and ureteroscopy including lithotripsy<br><br>Antibiotic selection based on urine culture susceptibility results, rectal swab, resistance risk

Assess Patient-Specific Risk for Antibiotic Resistance and Adverse Events

Did You Know ?

Prescribing discordant therapy (selection of the wrong antibiotic based on culture results) can increase risk of clinical failure.[4]

In a recent VA MEDSAFE utilization review[7] of 3,255 positive urine cultures collected in 31 VAMCs:

- 1 of 5 patients was prescribed discordant therapy
- Clinicians only selected the preferred antibiotic in approximately 50% of cases (preferred was defined using similar criteria described in this guide for ASB, cystitis, and pyelonephritis)

E.coli Susceptibilities (n=128[7])

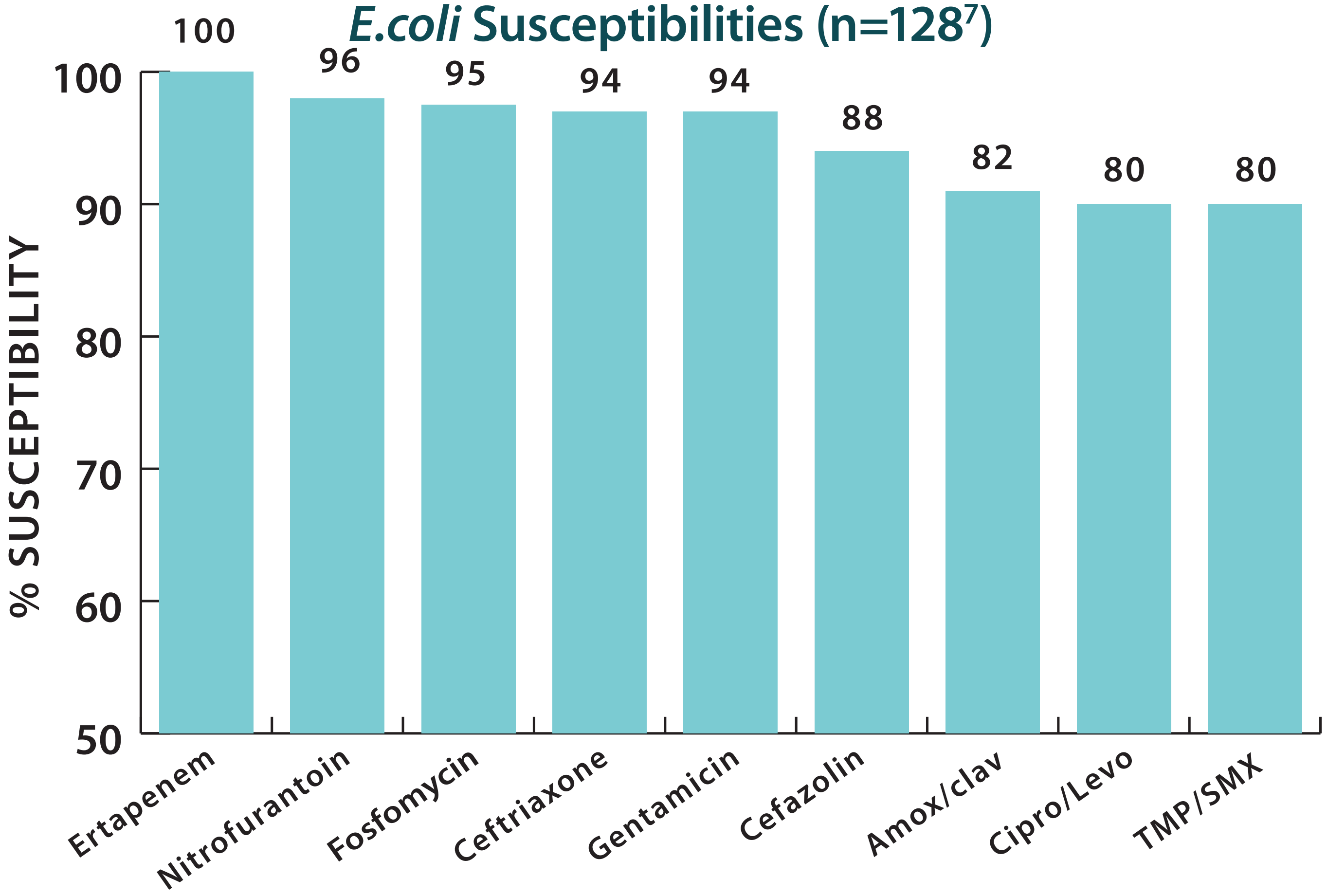

Considerations for selecting effective therapy: Antimicrobial spectrum

- Prior colonization with antibiotic resistant bacteria is a strong predictor of future antibiotic resistance.[7,11,12,14]

- Absence of antibiotic resistance risk factors (see table) indicates that an antibiotic resistant pathogen is unlikely.

Patient-specific factors	Risk of antibiotic resistant UTI
Antibiotic- resistant Gram-negative urinary isolate in prior 12 months	✓✓✓✓✓
Recent (3 months) specific antibiotic exposure (i.e. fluoroquinolones, sulfamethoxazole/ trimethoprim, 3rd generation cephalosporin)	✓✓✓✓
Recent inpatient stay (i.e. hospital, nursing home, long-term acute care facility)	✓✓✓✓
Recent travel abroad to a country with high antibiotic resistance (e.g. India, Israel, Spain, Mexico)	✓✓✓

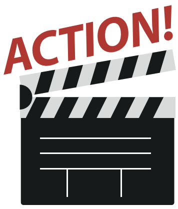

To prescribe concordant therapy:

- Obtain an accurate patient history including prior antibiotic treatments, microbiology results, hospitalizations, and travel.
- Avoid repeated courses of the same antibiotic and obtain a urine culture for patients with antibiotic resistance risk factors.

Considerations for selecting effective therapy: Safety

Antibiotic-related adverse events are common, accounting for 1 in 6 emergency department visits for adverse drug events.[15]

Pre-existing patient-specific health conditions or concurrent medications may exacerbate risk for harm.

Antibiotic	Common or serious adverse events	Patient factors that potentially increase risk for adverse outcomes
Nitrofurantoin	**Common:** nausea<br><br>**Serious:** interstitial pneumonitis, peripheral neuropathy, anemia	Renal impairment, advanced age (>65) , glucose-6-phosphatase dehydrogenase (G6PD) deficiency
Trimethoprim-sulfamethoxazole	**Common:** nausea, rash, drug interactions<br><br>**Serious:** hyperkalemia, Stevens-Johnson syndrome, toxic epidermal necrolysis, anemia, nephrotoxicity, hepatotoxicity	Renal impairment, advanced age (>65), pregnancy, folate or G6PD deficiency, thyroid dysfunction, concomitant use of ACEi/ARB/spironolactone/potassium, methotrexate, or warfarin
Cephalosporins/penicillins	**Common:** rash, *C. difficile*<br><br>**Serious:** anaphylaxis, anemia, leukopenia, hepatotoxicity	Prior type 1 hypersensitivity reaction (anaphylaxis, angioedema, hypotension, pruritis)
Fluoroquinolones	**Common:** *C. difficile*, drug-drug interactions<br><br>**Serious:** Tendon rupture, retinal detachment, aortic dissection, arrhythmia, dysglycemia, interstitial nephritis, CNS irritability or mental status changes	Prolonged QTc interval, renal impairment, myasthenia gravis, aortic aneurysm, advanced age (>65), diabetes, seizure or mental health disorders, concomitant use of antacids, NSAIDS, or theophylline
Fosfomycin	**Common:** diarrhea, nausea<br><br>**Serious:** pulmonary edema	Renal impairment

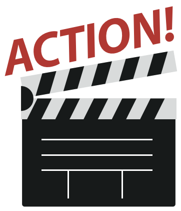

To prescribe the safest therapy:

- Obtain an accurate patient history including discussion of documented allergies, concurrent medications, and chronic diagnoses.
- When selecting an antibiotic, avoid agents with high potential for patient-specific harm.

Only Prescribe Fluoroquinolones in Absence of Appropriate Alternatives

? Did You Know

Antibiotics have different pharmacokinetic properties that can impact their effectiveness for UTIs depending on the anatomical location of infection.

Fluoroquinolones were once the preferred treatment for all UTIs due to their favorable genitourinary tract pharmacokinetic profile and broad Gram-negative coverage.

BUT, fluoroquinolones are no longer first line therapy for many UTIs

The Food and Drug Administration warns clinicians and patients not to use fluoroquinolones to treat uncomplicated UTIs due to multiple risks for serious adverse effects.[16]

Bacterial resistance to fluoroquinolones is now common in *E. coli* (see geographic figure below)

Fluoroquinolones also facilitate the transfer of antibiotic resistance genes in other bacteria and are risk factors for infection with ESBLs, CRE, MRSA, and *Clostridioides difficile*.

⚠ CAUTION

PRESCRIBE AT YOUR OWN RISK: FLUOROQUINOLONES

RESERVED FOR UTI w/LIMITED OPTIONS

FDA WARNINGS:
- Aortic Rupture/Aneurysm
- QTC Prolongation
- Tendon Rupture/Tendinitis
- Hypoglycemic Coma
- Peripheral Neuropathy
- Mental Health Side Effects

INCREASED RESISTANCE:
- ESBLs
- CRE
- MRSA
- *C.DIFFICILE*

ACTION!

To prescribe appropriate therapy

- Prescribe fluoroquinolones only when no other antibiotic alternatives exist

National and regional prevalence of ESBL phenotypes, levofloxacin- and trimethoprim-sulfamethoxazole-resistant phenotypes among 1831 isolates of E. coli from UTIs in the USA in 2017.[17]

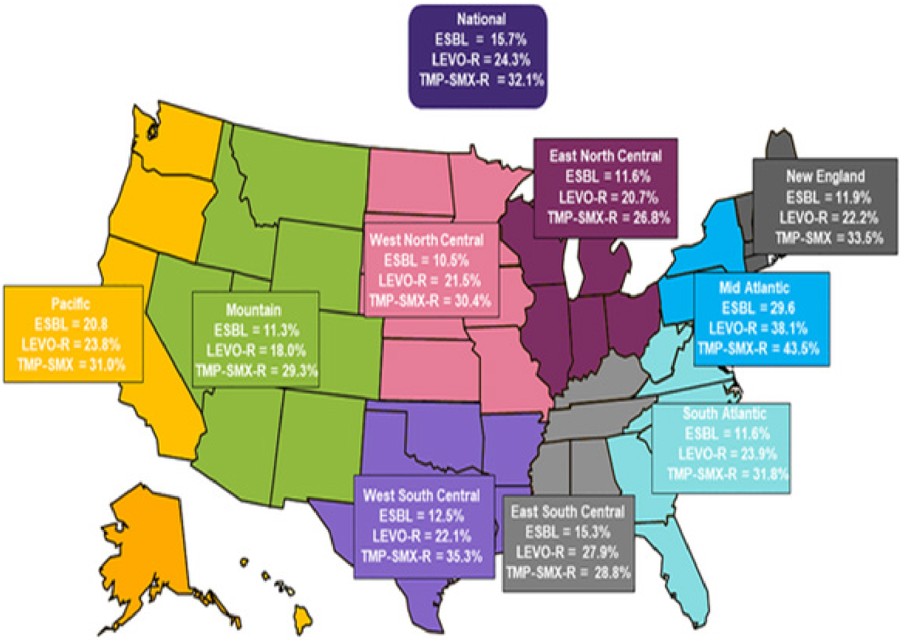

Optimal antibiotic prescribing for an individual patient requires:

- ✓ Identifying the most probable anatomical location of infection
- ✓ Assessing the risk for antibiotic resistance
- ✓ Avoiding antibiotics with high potential for patient-specific harm
- ✓ Prescribing the right dose and duration of therapy
- ✓ Follow-up on clinical improvement and susceptibilities

Table. Antibiotic Options and Duration of Therapy Recommendations for UTI[9,10,11,12]

UTI Diagnosis	Antibiotic Regimen	Comment
NOTE: Consider the initial administration of a one-time IM/IV dose of antibiotics followed by oral therapy depending on illness acuity and antibiotic resistance risk factors (ceftriaxone without risk factors; ertapenem or gentamicin with risk factors).		
**Women**		
**Acute Simple Cystitis**	Nitrofurantoin x 5 days<br><br>TMP/SMX x 3 days*<br><br>Cephalexin x 7 days*<br><br>Fosfomycin x 1 dose	Nitrofurantoin systemic or tissue concentrations insufficient to treat pyelonephritis<br><br>Fosfomycin less effective than other regimens. Availability may be dependent on local formulary. May be used for ESBL; Your local ID stewards will be able to assist in selecting the optimal antibiotic for ESBL.<br><br>*Cefpodoxime, cefdinir, cefadroxil, or TMP/SMX may be appropriate depending on local formulary and susceptibilities.
**Acute Pyelonephritis**	TMP/SMX x 7–14 days[18]*<br><br>Ciprofloxacin x 7 days<br><br>Levofloxacin x 5 days	Seek urology and/ or infectious diseases consultation for complicated cases.<br><br>Cephalosporins may be an alternative in women especially if they receive an initial dose parenteral antibiotics.<br><br>*TMP/SMX may be appropriate depending on susceptibilities; a longer duration (up to 14 days) of antibiotics may be necessary based on clinical response.

UTI Diagnosis	Antibiotic Regimen	Comment
NOTE: Consider the initial administration of a one-time IM/IV dose of antibiotics followed by oral therapy depending on illness acuity and antibiotic resistance risk factors (ceftriaxone without risk factors; ertapenem or gentamicin with risk factors).		
**Men**		
**Acute Simple Cystitis**	Nitrofurantoin x 7 days<br>TMP/SMX x 7 days*<br>Cephalexin x 7 days*	Nitrofurantoin systemic or tissue concentrations insufficient to treat pyelonephritis<br>For cystitis without pelvic or perineal pain or other findings suggestive of prostatitis<br>*Cefpodoxime, cefdinir, cefadroxil, or TMP/SMX may be appropriate depending on local formulary and susceptibilities.
**Acute Pyelonephritis**	TMP/SMX x 7–14 days days<br>Ciprofloxacin x 7 days<br>Levofloxacin x 5 days	Seek urology and/ or infectious diseases consultation for complicated cases or if pathogen is resistant to fluoroquinolones or TMP/SMX<br>*TMP/SMX may be appropriate depending on susceptibilities; a longer duration of antibiotics (up to 14 days) may be necessary based on clinical response (with the exception of fluoroquinolones).
**Acute Bacterial Prostatitis**	TMP/SMX x 14–42 days<br>Ciprofloxacin x 14–42 days<br>Levofloxacin x 14–42days	β-lactams and nitrofurantoin have limited effectiveness and should be avoided.<br>If sexually active, rule out gonorrhea and chlamydia; treat if positive.<br>Optimal treatment duration is unknown but clinical failure more common with <14 days of therapy<br>Seek urology and/ or infectious diseases consultation for complicated cases.
NOTE: Recommendations are for patients who are hemodynamically stable and able to tolerate oral medications.		

Treat Most UTIs for 7 Days or Less

Did You Know?

The correct duration of therapy is dependent upon patient sex, anatomical location of UTI, antibiotic selected; and for complicated infections, the clinical response to therapy.

Prescribing an antibiotic longer than recommended does not provide additional benefit even in uncomplicated pyelonephritis and Gram-negative bacteremia due to urosepsis.[19,20]

In the recent VA MEDSAFE utilization review[7] of 3,255 positive urine cultures collected in 31 VAMCs, clinicians treated for longer than recommended in more than 50% of patients.

Appropriate selection of patients for shortened antibiotic duration (≤7 days) for UTIs lead to fewer adverse events, and decreased risk of antibiotic resistance.

Before prescribing: Select the shortest duration of therapy consistent with infection anatomical location and antibiotic.

After prescribing: Follow up on culture results and patient

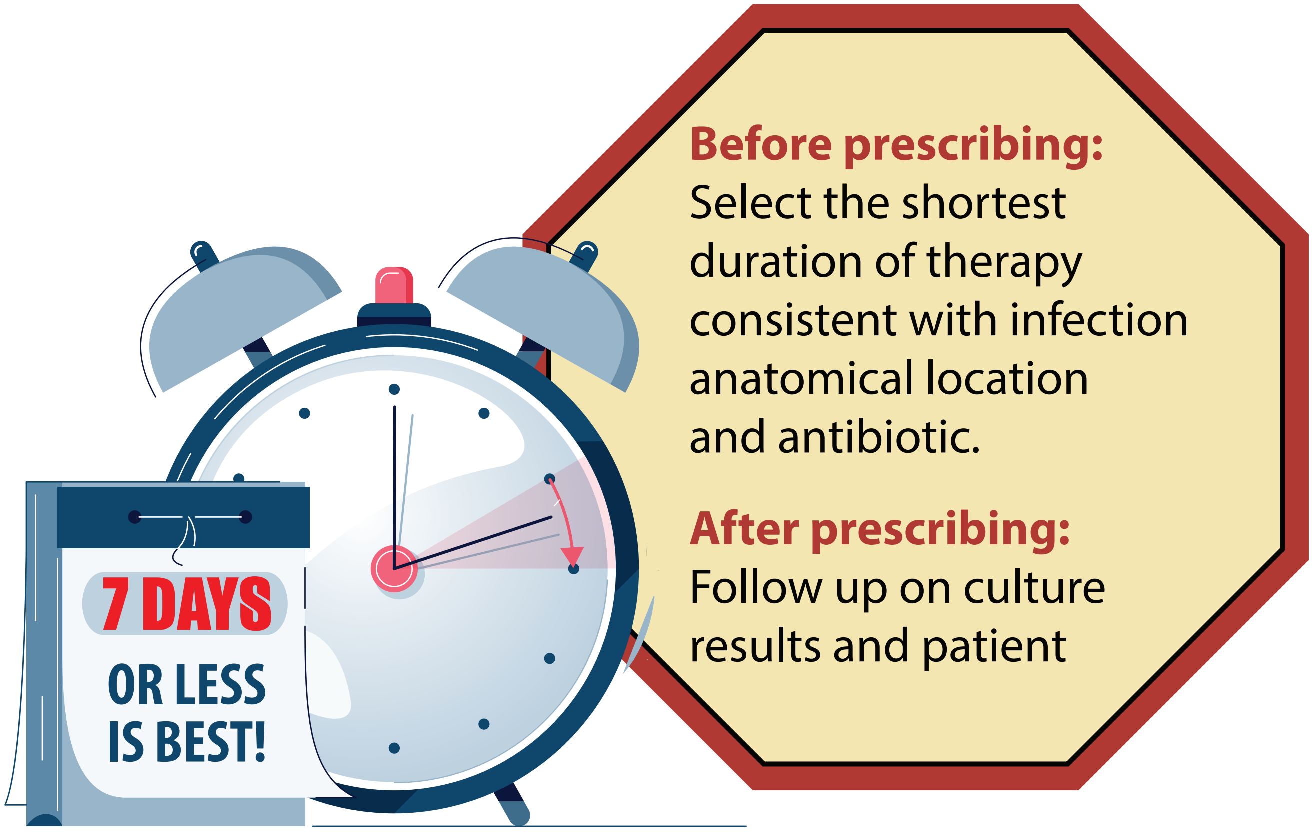

BIBLIOGRAPHY AND RESOURCES

1. Simmering JE, Tang F, Cavanaugh, et al. The increase in hospitalizations for urinary tract infections and the associated costs in the United States, 1998-2011. Open Forum Infect Dis. 2017 Winter; 4(1): ofw281. Doi. 10.1093/ofid/ofw281.

2. Nguyen HQ, Nguyen NTQ, Hughes CM, O'Neill C. Trends and impact of antimicrobial resistance on older inpatients with urinary tract infections (UTIs): A national retrospective observational study. 2019, PLoS ONE 14(10): e0223409. https://doi.org/10.1371/journal.pone.0223409

3. Goldstein E, MacFadden DR, Karaca Z, Steiner CA, Viboud C, Lipsitch M. Antimicrobial resistance prevalence, rates of hospitalization with septicemia and rates of mortality with sepsis in adults in different US states. Int J Antimicrob Agents. 2019 Jul;54(1):23-34. doi: 10.1016/j.ijantimicag.2019.03.004. Epub 2019 Mar 6. PMID: 30851403; PMCID: PMC6571064.

4. Talan DA, Takhar SS, Krishnadasan A, Mower WR, Pallin DJ, Garg M, Femling J, Rothman RE, Moore JC, Jones AE, Lovecchio F, Jui J, Steele MT, Stubbs AM, Chiang WK, Moran GJ. Emergence of Extended-Spectrum β-Lactamase Urinary Tract Infections Among Hospitalized Emergency Department Patients in the United States. Ann Emerg Med. 2021 Jan;77(1):32-43. doi: 10.1016/j.annemergmed.2020.08.022. Epub 2020 Oct 31. PMID: 33131912.

5. Cortes-Penfield NW, Trautner BW, Jump RL. Urinary tract infection and asymptomatic bacteriuria in older adults. Infect Dis Clin North Am. 2017;31(4):673-88.doi: 10.1016/j.idc.2017.07.002.

6. Anon. Reduce the rate of hospital admissions for urinary tract infections among older adults — OA 07. Health People 2030. Available at: https://health.gov/healthypeople/objectives-and-data/browse-objectives/infectious-disease/reduce-rate-hospital-admissions-urinary-tract-infections-among-older-adults-oa-07. Last accessed: February 23, 2021.

7. Urinary Tract Infections in the Outpatient Setting: Literature Review and Medication Utilization Review. Presented at the Monthly Antimicrobial Stewardship Taskforce (ASTF) Webinar series, October, 28th, 2020. VA MEDSafe, Data and protocol on file.

8. Schulz L, Hoffman RJ, Pothof J, Fox B. Top Ten Myths Regarding the Diagnosis and Treatment of Urinary Tract Infections. J Emerg Med. 2016 Jul;51(1):25-30. doi: 10.1016/j.jemermed.2016.02.009. Epub 2016 Apr 7. PMID: 27066953.

9. Gupta K, Hooton T, Naber K, Wullt B, Colgan R, Miller L, Moran G, Nicolle LE, Raz R, Schaeffer AJ, Soper D, International Clinical Practice Guidelines for the Treatment of Acute Uncomplicated Cystitis and Pyelonephritis in Women: A 2010 Update by the Infectious Diseases Society of America and the European Society for Microbiology and Infectious Diseases, Clinical Infectious Diseases, Volume 52, Issue 5, 1 March 2011, Pages e103–e120, https://doi.org/10.1093/cid/ciq257

10. Nichol LE, Gupta K, Bradley F, Colgan R, deMuri G, Drekonia D, Eckert L, Geerlines S, Kaves B, Hooton T, Juthani-Mehta, Knight S, Sait S, Schaeffer A, Trautner B, Wullt B, Siemleniuk. Clinical Practice Guideline for the Management of Asymptomatic Bacteriuria: 2019 Update by the Infectious Diseases Society of America, Clinical Infectious Diseases, Volume 68, Issue 10, 15 May 2019, Pages e83–e110, https://doi.org/10.1093/cid/ciy1121

11. Hooton T. Acute simple cystitis in men. Up to Date, 2020. Available At: https://www.uptodate.com/contents/acute-simple-cystitis-in-men. Last accessed: February 23, 2021

12. Hooton T, Gupta K. Acute complicated urinary tract infection (including pyelonephritis in adults. Up to Date, 2020. https://www.uptodate.com/contents/acute-complicated-urinary-tract-infection-including-pyelonephritis-in-adults?topicRef=8065&source=see_link. Last accessed: February 23, 2021

13. Zalmanovici Trestioreanu A, Lador A, Sauerbrun-Cutler MT, Leibovici L. Antibiotics for asymptomatic bacteriuria. Cochrane Database of Systematic Reviews 2015, Issue 4. Art. No.: CD009534. DOI: 10.1002/14651858.CD009534.pub2.

14. Linsenmeyer K, Strymish J, Gupta K. Two Simple Rules for Improving the Accuracy of Empiric Treatment of Multidrug-Resistant Urinary Tract Infections. Antimicrob Agents Chemother. 2015 Dec;59(12):7593-6. doi: 10.1128/AAC.01638-15. Epub 2015 Sep 28. PMID: 26416859; PMCID: PMC4649203.

15. Shehab N, Lovegrove MC, Geller AI, Rose KO, Weidle NJ, Budnitz DS. US Emergency Department Visits for Outpatient Adverse Drug Events, 2013-2014. JAMA. 2016;316(20):2115–2125. doi:10.1001/jama.2016.16201

16. FDA Drug Safety Communication: FDA advises restricting fluoroquinolone antibiotic use for certain uncomplicated infections; warns about disabling side effects that can occur together. Available at: https://www.fda.gov/drugs/drug-safety-and-availability/fda-drug-safety-communication-fda-advises-restricting-fluoroquinolone-antibiotic-use-certain. Last accessed February 23, 2021.

17. Critchley IA, Cotroneo N, Pucci MJ, Mendes R (2019) The burden of antimicrobial resistance among urinary tract isolates of Escherichia coli in the United States in 2017. PLOS ONE 14(12): e0220265. https://doi.org/10.1371/journal.pone.0220265

18. Fox MT, Melia MT, Same RG, Conley AT, Tamma PD. A seven-day course of tmp-smx may be as effective as a seven-day course of ciprofloxacin for the treatment of pyelonephritis. Am J Med. 2017;130(7):842-845.

19. Drekonja DM, Rector TS, Cutting A, Johnson JR. Urinary tract infection in male veterans: treatment patterns and outcomes. JAMA Intern Med. 2013 Jan 14;173(1):62-8. doi: 10.1001/2013.jamainternmed.829. PMID: 23212273.

20. Erickson RM, Tritle BJ, Spivak ES, Timbrook TT. Impact of an Antimicrobial Stewardship Bundle for Uncomplicated Gram-Negative Bacteremia. Open Forum Infect Dis. 2019 Nov 13;6(12):ofz490. doi: 10.1093/ofid/ofz490. PMID: 32128333; PMCID: PMC7047945.

Notes

Notes

Notes

Notes

Notes

U.S. Department of Veterans Affairs

This reference guide was created to be used as a tool for VA providers and is available to use from the Academic Detailing Service SharePoint.

These are general recommendations only; specific clinical decisions should be made by the treating provider based on an individual patient's clinical condition.

VISN21 Antimicrobial Stewardship Email Group and or ASTF Contact info:
VISN21ANTIMICROBIALSTEWARDSHIP@va.gov

VA ASTF SharePoint Site:
https://dvagov.sharepoint.com/sites/VHAPBM/ASTF/SitePages/ASTF.aspx

VA PBM Academic Detailing Service Email Group
PharmacyAcademicDetailingProgram@va.gov

VA PBM Academic Detailing Service SharePoint Site
https://dvagov.sharepoint.com/sites/vhaacademicdetailing/ClassicMigration/SitePages/Home.aspx

June 2021 IB 10-1638, P96879 **www.va.gov**
