# Supplementary material for: A pilot intervention to improve the management of urinary tract infections in outpatient settings
Source: Antimicrob Steward Healthc Epidemiol. 2025 Dec 18;5(1):e338. doi: 10.1017/ash.2025.10228 (PMC12722549; doi:10.1017/ash.2025.10228)
Supplement: Madaras-Kelly et al. supplementary material 2 — Madaras-Kelly et al. supplementary material [file S2732494X25102283sup002.pdf]

VA
U.S. Department of Veterans Affairs
Veterans Health Administration
PBM Academic Detailing Services

A QUICK REFERENCE GUIDE (2021)

Urinary Tract Infections

Appropriate Identification and Management of Urinary Tract Infections (UTIs) in the Outpatient Setting

VA PBM Academic Detailing Service

Real Provider Resources

Real Patient Results

Your Partner in Enhancing Veteran Health Outcomes

VISN21 Antimicrobial Stewardship Email Group and or ASTF Contact Info:

VISN21ANTIMICROBIALSTEWARDSHIP@va.gov

VA PBM Academic Detailing Service Email Group
PharmacyAcademicDetailingProgram@va.gov

VA PBM Academic Detailing Service SharePoint Site
https://vaww.portal2.va.gov/sites/ad

VA ASTF SharePoint Site: https://dvagov.sharepoint.com/sites/VHAPBM/ASTF/SitePages/ASTF.aspx

Table of Contents

Key Messages for Managing UTIs without Overusing Antibiotics 2

Treating Based on "UTI Misbeliefs" Can Lead to Incorrect Diagnosis and Antibiotic Overtreatment 3

Make a Specific Clinical UTI Diagnosis to Drive Appropriate Care 4

Differentiate Between Cystitis, Pyelonephritis, and Prostatitis and Utilize the Most Specific ICD-10 Code 8

Assess for Other Potential Etiologies of Urinary Symptoms 9

Prescribe Appropriate Selection and Duration of Antibiotic Therapy 10

Minimize Potential for Antibiotic Adverse Events 15

Treat Most UTIs for 7 Days or Less 17

Attention Healthcare Provider:

These recommendations are intended for treatment of immunocompetent patients with urinary tract infections (UTIs), and do not pertain to patients with epididymitis, orchitis, urinary diversions (e.g. ileal conduits, urinary stents, nephrostomy tubes), sexually transmitted infections, or other severe illnesses/impediments.

Individual patient-specific characteristics should be considered when determining appropriate therapy.

Key Messages for Managing UTIs without Overusing Antibiotics

Diagnostics

1. Avoid ordering urine cultures in the absence of UTI symptoms.
2. Urinalysis findings and altered mental status should not be used alone to establish a diagnosis of UTI.
3. Assess for other potential etiologies of urinary symptoms.
4. Differentiate between cystitis, pyelonephritis, and prostatitis; identify need for additional workup.

Treatment

1. Only treat asymptomatic bacteriuria (ASB) before invasive urological procedures or in pregnant patients.
2. Assess patient-specific risk for antibiotic resistance and adverse events.
3. Only prescribe fluoroquinolones in the absence of appropriate alternatives.
4. Treat most UTIs for 7 days or less.

Treating Based on "UTI Misbeliefs" Can Lead to Incorrect Diagnosis and Antibiotic Overtreatment

Unnecessarily treating asymptomatic bacteriuria (ASB) provides no additional clinical benefit—and increases risk of preventable adverse events.

ODOR ≠ "UTI"

Malodorus urine does not correlate well with infection and should not be considered a symptom of "UTI."

Cortes-Pinfield Infect Dis Clin North Am 2017 PMC 5802407

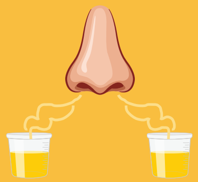

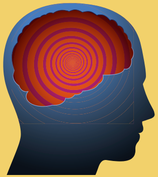

DELIRIUM ≠ "UTI"

- Don't blame "UTI"
- Perform a complex hx & exam
- Check pertinent labs & imaging
- Stop any potential culprit meds
- Ensure close follow up
- Send cultures and start empiric antibiotics if sick

The Curbsiders #134 Urinary Tract Infections Delirium and Voltaire

Antibiotic treatment of ASB is only appropriate in pregnancy (risk of preterm labor) and prior to select invasive urological procedures (risk of bacteremia +/- sepsis).

When should you NOT screen for and subsequently treat ASB?

- Premenopausal, nonpregnant women
- Patients with diabetes mellitus
- Older persons living in the community
- Elderly persons in long term care facilities
- Persons with spinal cord injury
- Catheterized patients while the catheter remains in situ

Make a Specific Clinical UTI Diagnosis to Drive Appropriate Care

Diagnosis	Signs/Symptoms	Antibiotic Treatment Recommendations
	Women	
**Acute Simple Cystitis**	**ONE or MORE of the following:**<br>Dysuria<br>Urinary frequency or urgency<br>Suprapubic pain<br>Acute gross hematuria<br>WITHOUT flank pain, CVA tenderness, rigors, chills, or subjective fever or temp. (<99.9F)	Bladder-concentrating antibiotics like nitrofurantoin can be utilized preferentially.<br>Shorter antibiotic durations can be utilized in most patients.

continued from page 4 (Make a Specific Clinical UTI Diagnosis to Drive Appropriate Care)

Diagnosis	Signs/Symptoms	Antibiotic Treatment Recommendations
	**Women**	
**Acute Pyelonephritis**	**ONE or MORE cystitis symptoms WITH at least ONE of the following:**<br>Flank pain<br>CVA tenderness<br>Rigors, chills, subjective fever or temp. (≥99.9F)	Some antibiotics (such as nitrofurantoin) should NOT be used as they don't reach adequate systemic or tissue concentrations.<br>Longer courses may be needed than for cystitis.

Presence of complicating conditions can increase the risk for recurrent infection or failing therapy. Complicated cases, patients with recurrent symptoms, or antibiotic resistant infections such as extended spectrum beta-lactamase producing organisms (ESBLs) may require referral to urology or infectious diseases (ID). Other reasons for urology referral include:

- history of bladder cancer
- history or risk of urethral strictures
- underlying neurologic disease that could be associated with a neurogenic bladder
- persistent or recurrent urinary retention (PVR >150 mL in older adults)

Urgent referral is indicated in the settings of renal compromise due to urinary retention, palpable bladder on exam or unexplained hematuria.

continued from page 5 (Make a Specific Clinical UTI Diagnosis to Drive Appropriate Care)

Diagnosis	Signs/Symptoms	Antibiotic Treatment Recommendations
	**Men**	
**Acute Simple Cystitis**	**ONE or MORE of the following:**<br>Dysuria<br>Urinary frequency or urgency<br>Suprapubic pain<br>Acute gross hematuria<br>WITHOUT the following: flank pain, CVA tenderness, pelvic or perineal pain, rigors, chills, or subjective fever or temp. (<99.9F)	Bladder-concentrating antibiotics, like nitrofurantoin, can be utilized preferentially<br>Duration of 7 days is adequate in most cases.
**Acute Pyelonephritis**	**ONE or MORE cystitis symptom WITH at least ONE of the following:**<br>Flank pain<br>CVA tenderness<br>Rigors, chills, subjective fever or temp. (<99.9F)	Some antibiotics (such as nitrofurantoin) should NOT be used as they don't reach adequate systemic or tissue concentrations.<br>Longer courses (up to 14 days) of antibiotics (with the exception of fluoroquinolones) may be needed for pyelonephritis.

continued from page 6 (Make a Specific Clinical UTI Diagnosis to Drive Appropriate Care)

Diagnosis	Signs/Symptoms	Antibiotic Treatment Recommendations
	**Men**	
**Acute Bacterial Prostatitis**	**ONE or MORE cystitis symptoms WITH at least ONE of the following:**<br>Enlarged firm tender prostate on digital rectal exam<br>Acute pelvic or perineal pain (especially with recurrent cystitis symptoms or obstructive symptoms- i.e. dribbling, hesitancy)<br>Rigors, chills, or subjective fever or temp. (≥99.9F)	Acute prostatitis will require longer courses of therapy; only select antibiotic classes should be used for prostatitis.

Presence of complicating conditions can increase the risk for recurrent infection or failing therapy. Complicated cases, patients with recurrent symptoms, or antibiotic resistant infections such as extended spectrum beta-lactamase producing organisms (ESBLs) may require referral to urology or infectious diseases (ID). Other reasons for urology referral include:

- history of prostate or bladder cancer
- history or risk of urethral strictures
- underlying neurologic disease that could be associated with a neurogenic bladder
- persistent or recurrent urinary retention (PVR >150 mL in older adults).

Urgent referral is indicated in the settings of renal compromise due to urinary retention, palpable bladder on exam or unexplained hematuria, or abnormal digital rectal exam findings that are suspicious for prostate cancer.

Differentiate between Cystitis, Pyelonephritis, and Prostatitis and Utilize the Most Specific ICD-10 Code

- The most common ICD10 code for UTIs is "Urinary tract infection, site not specified." It is recommended to differentiate the probable UTI subgroup diagnosis since treatment can vary according to the infection site.

Preferred UTI Codes	
**Acute Cystitis**	N30.0
**Acute Cystitis without Hematuria**	N30.00
**Acute Cystitis with Hematuria**	N30.01
**Acute Pyelonephritis**	N10.
**Acute Prostatitis**	N41.0
**Use R82.71 for asymptomatic patients. Use the following code for BPH with non-infectious symptoms**	
**Asymptomatic Bacteriuria**	R82.71
**Benign Prostatic Hyperplasia with Lower Urinary Tract Symptoms (≠ UTI)**	N40.1

Assess for Other Potential Etiologies of Urinary Symptoms

Symptom	Potential common non-infectious causes of symptoms often attributed to UTI
**Urinary Frequency/Urgency**	Benign prostatic hypertrophy, urethral stricture, overactive bladder, stress incontinence, overflow incontinence, excessive fluid/caffeine intake, diuretics, hyperglycemia
**Dysuria**	Urethral stricture, urothelial malignancy, sexually transmitted infection, urinary spasm, Candida vaginitis
**Suprapubic Pain/Pelvic Discomfort**	Urinary obstruction, constipation, diverticulitis, genitourinary malignancy, sexually transmitted infection, catheter misplacement, intra-abdominal etiology such as diverticulitis or appendicitis
**Flank Pain**	Renal infarct, malignancy, pleural pathology, nephrolithiasis
**Testicular Pain or Tenderness**	Epididymitis (unilateral pain), spermatic or testicular torsion
**Hematuria**	Urothelial/renal malignancy, nephrolithiasis, trauma, acute kidney injury, sexually transmitted infection excessive anticoagulant therapy, tuberculosis, sickle cell disease, chronic NSAID use
**Altered Mental Status**	Medication interactions, sundowning (especially with change in care setting), poor oral intake, electrolyte disturbance, trauma/head injury, stroke, CNS space-occupying lesion, infection elsewhere

Prescribe Appropriate Selection and Duration of Antibiotic Therapy

Step 1: Determine need for IV or IM one-time dose.

- Appropriate selection and duration of antibiotic therapy in UTIs—including reservation of fluoroquinolones unless there are no other alternatives.

§ If patient symptoms include high fever (>101°F), high leukocytosis, nausea/vomitting, structural or functional abnormality, immunosuppression, dehydration or evidence of sepsis consider hospital

¥ Complicated UTI risk factors for either men or women: indwelling urinary catheter, residual urine (>150 mL) after voiding (ex. urogenic bladder, benign prostatic hypertrophy), obstructive uropathy, or azotemia caused by intrinsic reanal disease.

* Your local ID stewards will be able to assist you in selecting the optimal IV or IM antibiotic based on local antibiogram and/or prior susceptibilities.

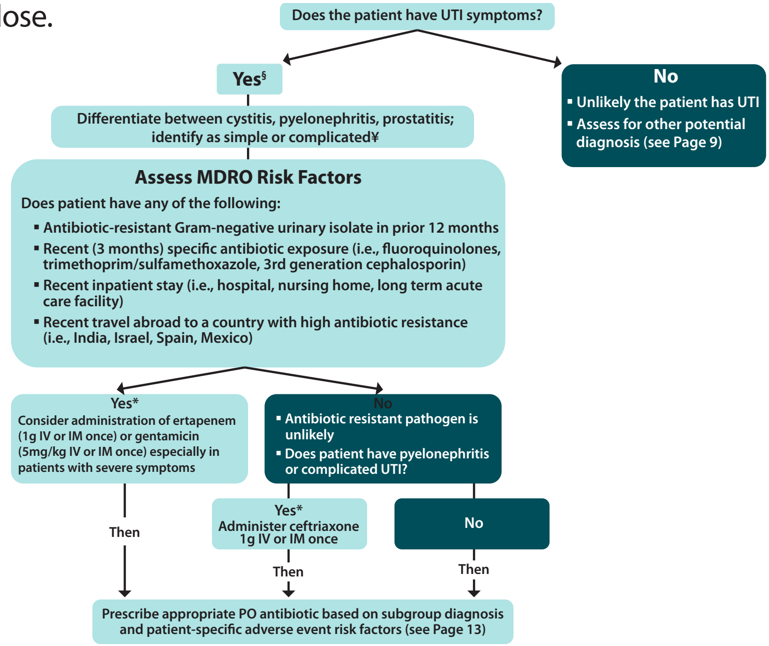

continued from page 10 (Prescribe Appropriate Selection and Duration of Antibiotic Therapy)

Step 2: Prescribe appropriate oral antibiotic with appropriate duration based on diagnosis

- These recommendations are based on patients with normal kidney function

Women		
**UTI Diagnosis**	**Antibiotic Regimen§**	**Comment**
**Acute Simple Cystitis**	Nitrofurantoin 100mg PO q12hrs x5 days<br>TMP/SMX 160mg/800mg PO q12hrs x3 days*<br>Cephalexin 500mg q6hrs x7 days*<br>Fosfomycin 3gm x1 dose	Nitrofurantoin systemic concentrations are insufficient to treat pyelonephritis; CrCl 30-60 mL/min: Although contraindicated in manufacturer labeling, limited data suggest nitrofurantoin is safe and effective for short-term treatment of uncomplicated acute cystitis in this patient population (Chung 2019; Cunha 2017; Oplinger 2013; Santos 2016; Singh 2015).<br>Fosfomycin less effective than other regimens. Availability may be dependent on local formulary. May be used for ESBL; Your local ID stewards will be able to assist in selecting the optimal antibiotic for ESBL.<br>*Cefpodoxime, cefdinir, cefadroxil, or TMP/SMX may be appropriate depending susceptibilities.

continued from page 11 (Prescribe Appropriate Selection and Duration of Antibiotic Therapy)

Women		
**UTI Diagnosis**	**Antibiotic Regimen[§]**	**Comment**
**Acute Pyelonephritis**	TMP/SMX 160mg/800mg PO q12hrs x7–14 days*<br><br>Ciprofloxacin 500mg PO q12hrs x7 days<br><br>Levofloxacin 750mg PO q24hrs x5 days	Seek urology and/or infectious diseases consultation for complicated cases.<br><br>Cephalosporins may be an alternative in women especially if they receive an initial dose of parental antibiotics (Am J Emerg Med 2018; 36(11):2054-57).<br><br>*TMP/SMX may be appropriate depending on susceptibilities; a longer duration (Up to 14 days) of antibiotics may be necessary based on clinical response (with the exception of fluoroquinolones).
Consider the initial administration of a one-time IM/IV dose of antibiotics depending on illness acuity and antibiotic resistance risk factors (ceftriaxone without risk factors, ertapenem, gentamicin with risk factors).		
[§]Antibiotic availability may be dependent upon local formulary		

continued from page 12 (Prescribe Appropriate Selection and Duration of Antibiotic Therapy)

Men		
**UTI Diagnosis**	**Antibiotic Regimen§**	**Comment**
**Acute Simple Cystitis**	Nitrofurantoin 100mg PO q12hrs x7 days<br>TMP/SMX 160mg/800mg PO q12hrs x7 days<br>Cephalexin 500mg q6hrs x7 days*	CrCl 30-60 mL/min: Although contraindicated in manufacturer labeling, limited data suggest nitrofurantoin is safe and effective for short-term treatment of uncomplicated acute cystitis in this patient population (Chung 2019; Cunha 2017; Oplinger 2013; Santos 2016; Singh 2015).<br>For cystitis W/O pelvic or perineal pain or other findings suggestive of prostatitis.<br>*Cefpodoxime, cefdinir, or cefadroxil, or TMP/SM may be appropriate depending on susceptibilities.
**Acute Pyelonephritis**	TMP/SMX 160mg/800mg PO q12hrs x7 days*<br>Ciprofloxacin 500mg q12hrs x7 days<br>Levofloxacin 750mg PO q24hrs x5 days	Seek urology consultation for complicated cases or infectious diseases if pathogen is resistant to TMP/SMX or fluoroquinolones<br>*TMP/SMX may be appropriate depending on susceptibilities; a longer duration of therapy (up to 14 days) may be necessary based on clinical response (with the exception of fluoroquinolones).

continued from page 13 (Prescribe Appropriate Selection and Duration of Antibiotic Therapy)

Men		
**UTI Diagnosis**	**Antibiotic Regimen[§]**	**Comment**
**Acute Bacterial Prostatitis**	TMP/SMX 160mg/800mg PO q12hrs x14–42 days<br><br>Ciprofloxacin 500mg q12 hrs x14–42 days<br><br>Levofloxacin 750mg PO q24hrs x14–42days	β-lactams and nitrofurantoin have poor prostate penetration and should be avoided.<br><br>If sexually active, R/O gonorrhea and chlamydia, treat if positive.<br><br>Optimal treatment duration is unknown but clinical failure more common with <14 days of therapy.<br><br>Seek urology and/ or infectious diseases consultation for complicated cases.
Consider the initial administration of a one-time IM/IV dose of antibiotics depending on illness acuity and antibiotic resistance risk factors (ceftriaxone without risk factors; ertapenem, gentamicin with risk factors).<br><br>[§]Antibiotic availability may be dependent upon local formulary.		

Minimize Potential for Antibiotic Adverse Events

Antibiotic	Common or Serious Adverse Events	Patient Factors that Potentially Increase Risk for Adverse Outcomes
**Nitrofurantoin**	**Common:** nausea<br>**Serious:** interstitial pneumonitis, peripheral neuropathy, anemia	Renal impairment, advanced age, glucose-6-phosphatase dehydrogenase (G6PD) deficiency
**Trimethoprim-sulfamethoxazole**	**Common:** nausea, rash, drug interactions<br>**Serious:** hyperkalemia, Stevens-Johnson syndrome, toxic epidermal necrolysis, anemia, nephrotoxicity, hepatotoxicity	Renal impairment, advanced age, pregnancy, folate or G6PD deficiency, thyroid dysfunction, concomitant use of ACEi/ARB/spironolactone/potassium, methotrexate, or warfarin
**Cephalosporins/ Penicillins**	**Common:** rash, C. difficile<br>**Serious:** anaphylaxis, anemia, leukopenia, hepatotoxicity	Prior type 1 hypersensitivity reaction (anaphylaxis, angioedema, hypotension, pruritis)

continued from page 15 (Minimize Potential for Antibiotic Adverse Events)

Antibiotic	Common or Serious Adverse Events	Patient Factors that Potentially Increase Risk for Adverse Outcomes
**Fluoroquinolones**	**Common:** C. difficile, drug-drug interactions<br>**Serious:** Tendon rupture, retinal detachment, aortic dissection, arrhythmia, dysglycemia, interstitial nephritis, CNS irritability or mental status changes	Prolonged QTc interval, renal impairment, myasthenia gravis, aortic aneurysm, advanced age, diabetes, seizure or mental health disorders, concomitant use of antacids, NSAIDS, or theophylline
**Fosfomycin**	**Common:** diarrhea, nausea<br>**Serious:** pulmonary edema	Renal impairment

Treat Most UTIs For 7 Days or Less

Before prescribing:
Select the shortest duration of therapy consistent with infection anatomical location and antibiotic.

After prescribing:
Follow up on culture results and patient

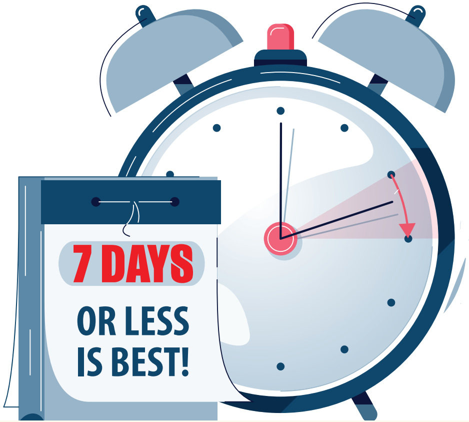

The correct duration of therapy is dependent upon patient sex, anatomical location of UTI, antibiotic selected; and for complicated infections, the clinical response to therapy.

U.S. Department of Veterans Affairs

This reference guide was created to be used as a tool for VA providers and is available to use from the Academic Detailing SharePoint. These are general recommendations only; specific clinical decisions should be made by the treating provider based on an individual patient's clinical condition.

VA PBM Academic Detailing Service Email Group
PharmacyAcademicDetailingProgram@va.gov

VA PBM Academic Detailing Service SharePoint Site
https://vaww.portal2.va.gov/sites/ad/SitePages/Home.aspx

VA PBM Academic Detailing Public Website
http://www.pbm.va.gov/PBM/academicdetailingservicehome.asp

June 2021

IB 10-1639, P97086

www.va.gov
