# Supplementary material for: A pilot intervention to improve the management of urinary tract infections in outpatient settings
Source: Antimicrob Steward Healthc Epidemiol. 2025 Dec 18;5(1):e338. doi: 10.1017/ash.2025.10228 (PMC12722549; doi:10.1017/ash.2025.10228)
Supplement: Madaras-Kelly et al. supplementary material 3 — Madaras-Kelly et al. supplementary material [file S2732494X25102283sup003.docx]

**Supplement 2: Inclusion& Exclusion ICD-10 CM Codes**

| **ICD10Code** | **ICD10Description** | **Final Tier** | **UTI** | **Cystitis** | **Epididymitis** | **Pyelonephritis** |
| --- | --- | --- | --- | --- | --- | --- |
| N10. | Acute pyelonephritis | 1 | 1 | 0 | 0 | 1 |
| N11.0 | Nonobstructive reflux-associated chronic pyelonephritis | 2 | 0 | 0 | 0 | 0 |
| N11.1 | Chronic obstructive pyelonephritis | 2 | 0 | 0 | 0 | 0 |
| N11.8 | Other chronic tubulo-interstitial nephritis | 2 | 0 | 0 | 0 | 0 |
| N11.9 | Chronic tubulo-interstitial nephritis, unspecified | 2 | 0 | 0 | 0 | 0 |
| N12. | Tubulo-interstitial nephritis, not specified as acute or chronic | 2 | 0 | 0 | 0 | 0 |
| N13.1 | Hydronephrosis with ureteral stricture, not elsewhere classified | 3 | 0 | 0 | 0 | 0 |
| N13.2 | Hydronephrosis with renal and ureteral calculous obstruction | 3 | 0 | 0 | 0 | 0 |
| N13.30 | Unspecified hydronephrosis | 3 | 0 | 0 | 0 | 0 |
| N13.39 | Other hydronephrosis | 3 | 0 | 0 | 0 | 0 |
| N13.6 | Pyonephrosis | 1 | 0 | 0 | 0 | 0 |
| N13.70 | Vesicoureteral-reflux, unspecified | 2 | 0 | 0 | 0 | 0 |
| N13.71 | Vesicoureteral-reflux without reflux nephropathy | 2 | 0 | 0 | 0 | 0 |
| N13.721 | Vesicoureteral-reflux with reflux nephropathy without hydroureter, unilateral | 2 | 0 | 0 | 0 | 0 |
| N13.722 | Vesicoureteral-reflux with reflux nephropathy without hydroureter, bilateral | 2 | 0 | 0 | 0 | 0 |
| N13.729 | Vesicoureteral-reflux with reflux nephropathy without hydroureter, unspecified | 2 | 0 | 0 | 0 | 0 |
| N13.731 | Vesicoureteral-reflux with reflux nephropathy with hydroureter, unilateral | 2 | 0 | 0 | 0 | 0 |
| N13.732 | Vesicoureteral-reflux with reflux nephropathy with hydroureter, bilateral | 2 | 0 | 0 | 0 | 0 |
| N13.739 | Vesicoureteral-reflux with reflux nephropathy with hydroureter, unspecified | 2 | 0 | 0 | 0 | 0 |
| N13.8 | Other obstructive and reflux uropathy | 3 | 0 | 0 | 0 | 0 |
| N13.9 | Obstructive and reflux uropathy, unspecified | 3 | 0 | 0 | 0 | 0 |
| N15.1 | Renal and perinephric abscess | 1 | 1 | 0 | 0 | 1 |
| N15.8 | Other specified renal tubulo-interstitial diseases | 2 | 0 | 0 | 0 | 0 |
| N15.9 | Renal tubulo-interstitial disease, unspecified | 2 | 0 | 0 | 0 | 0 |
| N16. | Renal tubulo-interstitial disorders in diseases classified elsewhere | 2 | 0 | 0 | 0 | 0 |
| N17.8 | Other acute kidney failure | 3 | 0 | 0 | 0 | 0 |
| N17.9 | Acute kidney failure, unspecified | 3 | 0 | 0 | 0 | 0 |
| N19. | Unspecified kidney failure | 3 | 0 | 0 | 0 | 0 |
| N20.0 | Calculus of kidney | 3 | 0 | 0 | 0 | 0 |
| N20.1 | Calculus of ureter | 3 | 0 | 0 | 0 | 0 |
| N20.2 | Calculus of kidney with calculus of ureter | 3 | 0 | 0 | 0 | 0 |
| N20.9 | Urinary calculus, unspecified | 3 | 0 | 0 | 0 | 0 |
| N21.0 | Calculus in bladder | 3 | 0 | 0 | 0 | 0 |
| N21.1 | Calculus in urethra | 3 | 0 | 0 | 0 | 0 |
| N21.8 | Other lower urinary tract calculus | 3 | 0 | 0 | 0 | 0 |
| N21.9 | Calculus of lower urinary tract, unspecified | 3 | 0 | 0 | 0 | 0 |
| N23. | Unspecified renal colic | 3 | 0 | 0 | 0 | 0 |
| N28.1 | Cyst of kidney, acquired | 3 | 0 | 0 | 0 | 0 |
| N28.84 | Pyelitis cystica | 2 | 0 | 0 | 0 | 0 |
| N28.85 | Pyeloureteritis cystica | 2 | 0 | 0 | 0 | 0 |
| N28.86 | Ureteritis cystica | 2 | 0 | 0 | 0 | 0 |
| N28.89 | Other specified disorders of kidney and ureter | 3 | 0 | 0 | 0 | 0 |
| N28.9 | Disorder of kidney and ureter, unspecified | 3 | 0 | 0 | 0 | 0 |
| N30.00 | Acute cystitis without hematuria | 1 | 1 | 1 | 0 | 0 |
| N30.01 | Acute cystitis with hematuria | 1 | 1 | 1 | 0 | 0 |
| N30.10 | Interstitial cystitis (chronic) without hematuria | 3 | 0 | 0 | 0 | 0 |
| N30.11 | Interstitial cystitis (chronic) with hematuria | 3 | 0 | 0 | 0 | 0 |
| N30.20 | Other chronic cystitis without hematuria | 2 | 0 | 0 | 0 | 0 |
| N30.21 | Other chronic cystitis with hematuria | 2 | 0 | 0 | 0 | 0 |
| N30.40 | Irradiation cystitis without hematuria | 3 | 0 | 0 | 0 | 0 |
| N30.41 | Irradiation cystitis with hematuria | 3 | 0 | 0 | 0 | 0 |
| N30.80 | Other cystitis without hematuria | 2 | 0 | 0 | 0 | 0 |
| N30.81 | Other cystitis with hematuria | 2 | 0 | 0 | 0 | 0 |
| N30.90 | Cystitis, unspecified without hematuria | 2 | 0 | 0 | 0 | 0 |
| N30.91 | Cystitis, unspecified with hematuria | 2 | 0 | 0 | 0 | 0 |
| N31.8 | Other neuromuscular dysfunction of bladder | 3 | 0 | 0 | 0 | 0 |
| N31.9 | Neuromuscular dysfunction of bladder, unspecified | 3 | 0 | 0 | 0 | 0 |
| N32.0 | Bladder-neck obstruction | 3 | 0 | 0 | 0 | 0 |
| N32.1 | Vesicointestinal fistula | 2 | 0 | 0 | 0 | 0 |
| N32.81 | Overactive bladder | 3 | 0 | 0 | 0 | 0 |
| N32.89 | Other specified disorders of bladder | 3 | 0 | 0 | 0 | 0 |
| N32.9 | Bladder disorder, unspecified | 3 | 0 | 0 | 0 | 0 |
| N33. | Bladder disorders in diseases classified elsewhere | 3 | 0 | 0 | 0 | 0 |
| N34.0 | Urethral abscess | 1 | 0 | 0 | 0 | 0 |
| N34.1 | Nonspecific urethritis | 2 | 0 | 0 | 0 | 0 |
| N34.2 | Other urethritis | 2 | 0 | 0 | 0 | 0 |
| N35.010 | Post-traumatic urethral stricture, male, meatal | 3 | 0 | 0 | 0 | 0 |
| N35.013 | Post-traumatic anterior urethral stricture | 3 | 0 | 0 | 0 | 0 |
| N35.014 | Post-traumatic urethral stricture, male, unspecified | 3 | 0 | 0 | 0 | 0 |
| N35.119 | Postinfective urethral stricture, not elsewhere classified, male, unspecified | 3 | 0 | 0 | 0 | 0 |
| N35.8 | Other urethral stricture | 3 | 0 | 0 | 0 | 0 |
| N35.811 | Other urethral stricture, male, meatal | 3 | 0 | 0 | 0 | 0 |
| N35.812 | Other urethral bulbous stricture, male | 3 | 0 | 0 | 0 | 0 |
| N35.813 | Other membranous urethral stricture, male | 3 | 0 | 0 | 0 | 0 |
| N35.819 | Other urethral stricture, male, unspecified site | 3 | 0 | 0 | 0 | 0 |
| N35.9 | Urethral stricture, unspecified | 3 | 0 | 0 | 0 | 0 |
| N35.912 | Unspecified bulbous urethral stricture, male | 3 | 0 | 0 | 0 | 0 |
| N35.913 | Unspecified membranous urethral stricture, male | 3 | 0 | 0 | 0 | 0 |
| N35.914 | Unspecified anterior urethral stricture, male | 3 | 0 | 0 | 0 | 0 |
| N35.919 | Unspecified urethral stricture, male, unspecified site | 3 | 0 | 0 | 0 | 0 |
| N39.0 | Urinary tract infection, site not specified | 1 | 1 | 1 | 0 | 0 |
| N39.3 | Stress incontinence (female) (male) | 3 | 0 | 0 | 0 | 0 |
| N39.41 | Urge incontinence | 3 | 0 | 0 | 0 | 0 |
| N39.46 | Mixed incontinence | 3 | 0 | 0 | 0 | 0 |
| N39.498 | Other specified urinary incontinence | 3 | 0 | 0 | 0 | 0 |
| N39.8 | Other specified disorders of urinary system | 3 | 0 | 0 | 0 | 0 |
| N39.9 | Disorder of urinary system, unspecified | 3 | 0 | 0 | 0 | 0 |
| N40.0 | Benign prostatic hyperplasia without lower urinary tract symptoms | 3 | 0 | 0 | 0 | 0 |
| N40.1 | Benign prostatic hyperplasia with lower urinary tract symptoms | 3 | 0 | 0 | 0 | 0 |
| N40.2 | Nodular prostate without lower urinary tract symptoms | 3 | 0 | 0 | 0 | 0 |
| N40.3 | Nodular prostate with lower urinary tract symptoms | 3 | 0 | 0 | 0 | 0 |
| N41.0 | Acute prostatitis | 1 | 0 | 0 | 0 | 0 |
| N41.1 | Chronic prostatitis | 2 | 0 | 0 | 0 | 0 |
| N41.2 | Abscess of prostate | 1 | 0 | 0 | 0 | 0 |
| N41.3 | Prostatocystitis | 2 | 0 | 0 | 0 | 0 |
| N41.4 | Granulomatous prostatitis | 2 | 0 | 0 | 0 | 0 |
| N41.8 | Other inflammatory diseases of prostate | 2 | 0 | 0 | 0 | 0 |
| N41.9 | Inflammatory disease of prostate, unspecified | 2 | 0 | 0 | 0 | 0 |
| N42.89 | Other specified disorders of prostate | 3 | 0 | 0 | 0 | 0 |
| N42.9 | Disorder of prostate, unspecified | 3 | 0 | 0 | 0 | 0 |
| N43.1 | Infected hydrocele | 1 | 0 | 0 | 0 | 0 |
| N43.3 | Hydrocele, unspecified | 3 | 0 | 0 | 0 | 0 |
| N45.1 | Epididymitis | 2 | 0 | 0 | 1 | 0 |
| N45.2 | Orchitis | 2 | 0 | 0 | 1 | 0 |
| N45.3 | Epididymo-orchitis | 2 | 0 | 0 | 1 | 0 |
| N45.4 | Abscess of epididymis or testis | 1 | 0 | 0 | 0 | 0 |
| N46.022 | Azoospermia due to infection | 2 | 0 | 0 | 0 | 0 |
| N46.025 | Azoospermia due to systemic disease | 2 | 0 | 0 | 0 | 0 |
| N46.122 | Oligospermia due to infection | 2 | 0 | 0 | 0 | 0 |
| N46.125 | Oligospermia due to systemic disease | 2 | 0 | 0 | 0 | 0 |
| N47.1 | Phimosis | 2 | 0 | 0 | 0 | 0 |
| N47.2 | Paraphimosis | 1 | 0 | 0 | 0 | 0 |
| N47.6 | Balanoposthitis | 2 | 0 | 0 | 0 | 0 |
| N48.1 | Balanitis | 2 | 0 | 0 | 0 | 0 |
| N48.21 | Abscess of corpus cavernosum and penis | 1 | 0 | 0 | 0 | 0 |
| N48.22 | Cellulitis of corpus cavernosum and penis | 1 | 0 | 0 | 0 | 0 |
| N48.29 | Other inflammatory disorders of penis | 2 | 0 | 0 | 0 | 0 |
| N48.5 | Ulcer of penis | 2 | 0 | 0 | 0 | 0 |
| N49.0 | Inflammatory disorders of seminal vesicle | 2 | 0 | 0 | 0 | 0 |
| N49.1 | Inflammatory disorders of spermatic cord, tunica vaginalis and vas deferens | 2 | 0 | 0 | 0 | 0 |
| N49.2 | Inflammatory disorders of scrotum | 2 | 0 | 0 | 0 | 0 |
| N49.3 | Fournier gangrene | 1 | 0 | 0 | 0 | 0 |
| N49.8 | Inflammatory disorders of other specified male genital organs | 2 | 0 | 0 | 0 | 0 |
| N49.9 | Inflammatory disorder of unspecified male genital organ | 2 | 0 | 0 | 0 | 0 |
| N50.8 | Other specified Disorders of Male Genital Organs | 3 | 0 | 0 | 0 | 0 |
| N50.811 | Right testicular pain | 2 | 0 | 0 | 0 | 0 |
| N50.812 | Left testicular pain | 2 | 0 | 0 | 0 | 0 |
| N50.819 | Testicular pain, unspecified | 2 | 0 | 0 | 0 | 0 |
| N50.82 | Scrotal pain | 2 | 0 | 0 | 0 | 0 |
| N70.01 | Acute salpingitis | 1 | 0 | 0 | 0 | 0 |
| N70.02 | Acute oophoritis | 1 | 0 | 0 | 0 | 0 |
| N70.03 | Acute salpingitis and oophoritis | 1 | 0 | 0 | 0 | 0 |
| N70.11 | Chronic salpingitis | 1 | 0 | 0 | 0 | 0 |
| N70.12 | Chronic oophoritis | 1 | 0 | 0 | 0 | 0 |
| N70.13 | Chronic salpingitis and oophoritis | 1 | 0 | 0 | 0 | 0 |
| N70.91 | Salpingitis, unspecified | 1 | 0 | 0 | 0 | 0 |
| N70.92 | Oophoritis, unspecified | 1 | 0 | 0 | 0 | 0 |
| N70.93 | Salpingitis and oophoritis, unspecified | 1 | 0 | 0 | 0 | 0 |
| N71.0 | Acute inflammatory disease of uterus | 2 | 0 | 0 | 0 | 0 |
| N71.1 | Chronic inflammatory disease of uterus | 2 | 0 | 0 | 0 | 0 |
| N71.9 | Inflammatory disease of uterus, unspecified | 2 | 0 | 0 | 0 | 0 |
| N72. | Inflammatory disease of cervix uteri | 2 | 0 | 0 | 0 | 0 |
| N73.0 | Acute parametritis and pelvic cellulitis | 1 | 0 | 0 | 0 | 0 |
| N73.1 | Chronic parametritis and pelvic cellulitis | 1 | 0 | 0 | 0 | 0 |
| N73.2 | Unspecified parametritis and pelvic cellulitis | 1 | 0 | 0 | 0 | 0 |
| N73.3 | Female acute pelvic peritonitis | 1 | 0 | 0 | 0 | 0 |
| N73.4 | Female chronic pelvic peritonitis | 1 | 0 | 0 | 0 | 0 |
| N73.5 | Female pelvic peritonitis, unspecified | 1 | 0 | 0 | 0 | 0 |
| N73.8 | Other specified female pelvic inflammatory diseases | 1 | 0 | 0 | 0 | 0 |
| N73.9 | Female pelvic inflammatory disease, unspecified | 1 | 0 | 0 | 0 | 0 |
| N74. | Female pelvic inflammatory disorders in diseases classified elsewhere | 1 | 0 | 0 | 0 | 0 |
| N75.1 | Abscess of Bartholin's gland | 2 | 0 | 0 | 0 | 0 |
| N75.8 | Other diseases of Bartholin's gland | 2 | 0 | 0 | 0 | 0 |
| N75.9 | Disease of Bartholin's gland, unspecified | 2 | 0 | 0 | 0 | 0 |
| N76.0 | Acute vaginitis | 2 | 0 | 0 | 0 | 0 |
| N76.1 | Subacute and chronic vaginitis | 2 | 0 | 0 | 0 | 0 |
| N76.2 | Acute vulvitis | 2 | 0 | 0 | 0 | 0 |
| N76.3 | Subacute and chronic vulvitis | 2 | 0 | 0 | 0 | 0 |
| N76.4 | Abscess of vulva | 2 | 0 | 0 | 0 | 0 |
| N76.5 | Ulceration of vagina | 2 | 0 | 0 | 0 | 0 |
| N76.6 | Ulceration of vulva | 2 | 0 | 0 | 0 | 0 |
| N76.81 | Mucositis (ulcerative) of vagina and vulva | 2 | 0 | 0 | 0 | 0 |
| N76.89 | Other specified inflammation of vagina and vulva | 2 | 0 | 0 | 0 | 0 |
| N77.0 | Ulceration of vulva in diseases classified elsewhere | 2 | 0 | 0 | 0 | 0 |
| N77.1 | Vaginitis, vulvitis and vulvovaginitis in diseases classified elsewhere | 2 | 0 | 0 | 0 | 0 |
| N82.2 | Fistula of vagina to small intestine | 2 | 0 | 0 | 0 | 0 |
| N82.3 | Fistula of vagina to large intestine | 2 | 0 | 0 | 0 | 0 |
| N82.4 | Other female intestinal-genital tract fistulae | 2 | 0 | 0 | 0 | 0 |
| N93.9 | Abnormal uterine and vaginal bleeding, unspecified | 3 | 0 | 0 | 0 | 0 |
| N98.0 | Infection associated with artificial insemination | 2 | 0 | 0 | 0 | 0 |
| N99.114 | Postprocedural urethral stricture, male, unspecified | 3 | 0 | 0 | 0 | 0 |
| N99.511 | Cystostomy infection | 1 | 0 | 0 | 0 | 0 |
| N99.521 | Infection of incontinent external stoma of urinary tract | 1 | 0 | 0 | 0 | 0 |
| N99.531 | INFECTION OF OTHER STOMA OF URINARY TRACT | 1 | 0 | 0 | 0 | 0 |
| N99.71 | Accidental puncture and laceration of a genitourinary system organ or structure during a genitourinary system procedure | 2 | 0 | 0 | 0 | 0 |
| N99.72 | Accidental puncture and laceration of a genitourinary system organ or structure during other procedure | 2 | 0 | 0 | 0 | 0 |
| R30.0 | Dysuria | 2 | 0 | 0 | 0 | 0 |
| R30.9 | Painful micturition, unspecified | 2 | 0 | 0 | 0 | 0 |
| R31.0 | Gross hematuria | 2 | 0 | 0 | 0 | 0 |
| R31.1 | Benign essential microscopic hematuria | 2 | 0 | 0 | 0 | 0 |
| R31.2 | Other Microscopic Hematuria | 3 | 0 | 0 | 0 | 0 |
| R31.21 | Asymptomatic microscopic hematuria | 3 | 0 | 0 | 0 | 0 |
| R31.29 | Other microscopic hematuria | 2 | 0 | 0 | 0 | 0 |
| R31.9 | Hematuria, unspecified | 2 | 0 | 0 | 0 | 0 |
| R32. | Unspecified urinary incontinence | 2 | 0 | 0 | 0 | 0 |
| R33.8 | Other retention of urine | 3 | 0 | 0 | 0 | 0 |
| R33.9 | Retention of urine, unspecified | 3 | 0 | 0 | 0 | 0 |
| R34. | Anuria and oliguria | 3 | 0 | 0 | 0 | 0 |
| R35.0 | Frequency of micturition | 3 | 0 | 0 | 0 | 0 |
| R35.1 | Nocturia | 3 | 0 | 0 | 0 | 0 |
| R35.8 | Other polyuria | 3 | 0 | 0 | 0 | 0 |
| R36.0 | Urethral discharge without blood | 2 | 0 | 0 | 0 | 0 |
| R36.9 | Urethral discharge, unspecified | 2 | 0 | 0 | 0 | 0 |
| R39.11 | Hesitancy of micturition | 3 | 0 | 0 | 0 | 0 |
| R39.14 | Feeling of incomplete bladder emptying | 3 | 0 | 0 | 0 | 0 |
| R39.15 | Urgency of urination | 2 | 0 | 0 | 0 | 0 |
| R39.19 | Other Difficulties with Micturition | 3 | 0 | 0 | 0 | 0 |
| R39.191 | Need to immediately re-void | 2 | 0 | 0 | 0 | 0 |
| R39.198 | Other difficulties with micturition | 3 | 0 | 0 | 0 | 0 |
| R39.81 | Functional urinary incontinence | 3 | 0 | 0 | 0 | 0 |
| R39.82 | Chronic bladder pain | 3 | 0 | 0 | 0 | 0 |
| R39.89 | Other symptoms and signs involving the genitourinary system | 3 | 0 | 0 | 0 | 0 |
| R50.81 | Fever presenting with conditions classified elsewhere | 2 | 0 | 0 | 0 | 0 |
| R50.9 | Fever, unspecified | 2 | 0 | 0 | 0 | 0 |
| R82.71 | Bacteriuria | 2 | 0 | 0 | 0 | 0 |
| R82.79 | Other abnormal findings on microbiological examination of urine | 2 | 0 | 0 | 0 | 0 |
| R82.81 | Pyuria | 2 | 0 | 0 | 0 | 0 |

**Supplement 1: Exclusion ICD-10 CM Codes**

| **ICDCode** | **ICDDescription** |
| --- | --- |
| 344 | QUADRIPLEGIA, UNSPECIFIED |
| 344 | QUADRIPLEGIA, UNSPECIFIED |
| 344.01 | QUADRIPLEGIA, C1-C4, COMPLETE |
| 344.02 | QUADRIPLEGIA, C1-C4, INCOMPLETE |
| 344.03 | QUADRIPLEGIA, C5-C7, COMPLETE |
| 344.04 | QUADRIPLEGIA, C5-C7, INCOMPLETE |
| 344.09 | OTHER QUADRIPLEGIA |
| 344.1 | PARAPLEGIA |
| 996.81 | COMPLICATIONS OF TRANSPLANTED KIDNEY |
| 996.82 | COMPLICATIONS OF TRANSPLANTED LIVER |
| 996.83 | COMPLICATIONS OF TRANSPLANTED HEART |
| 996.84 | COMPLICATIONS OF TRANSPLANTED LUNG |
| 996.85 | COMPLICATIONS OF BONE MARROW TRANSPLANT |
| 996.86 | COMPLICATIONS OF TRANSPLANTED PANCREAS |
| 996.87 | COMPLICATIONS OF TRANSPLANTED ORGAN,INTESTINE |
| G82.21 | Paraplegia, complete |
| G82.22 | Paraplegia, incomplete |
| G82.50 | Quadriplegia, unspecified |
| G82.51 | Quadriplegia, C1-C4 complete |
| G82.52 | Quadriplegia, C1-C4 incomplete |
| G82.53 | Quadriplegia, C5-C7 complete |
| G82.54 | Quadriplegia, C5-C7 incomplete |
| T86.00 | Unspecified complication of bone marrow transplant |
| T86.01 | Bone marrow transplant rejection |
| T86.02 | Bone marrow transplant failure |
| T86.09 | Other complications of bone marrow transplant |
| T86.10 | Unspecified complication of kidney transplant |
| T86.11 | Kidney transplant rejection |
| T86.12 | Kidney transplant failure |
| T86.20 | Unspecified complication of heart transplant |
| T86.21 | Heart transplant rejection |
| T86.22 | Heart transplant failure |
| T86.40 | Unspecified complication of liver transplant |
| T86.41 | Liver transplant rejection |
| T86.42 | Liver transplant failure |
| T86.810 | Lung transplant rejection |
| T86.811 | Lung transplant failure |
| T86.819 | Unspecified complication of lung transplant |
| T86.850 | Intestine transplant rejection |
| T86.851 | Intestine transplant failure |
| T86.859 | Unspecified complication of intestine transplant |
| T86.890 | Other transplanted tissue rejection |
| T86.891 | Other transplanted tissue failure |
| T86.899 | Unspecified complication of other transplanted tissue |
| V42.0 | KIDNEY REPLACED BY TRANSPLANT |
| V42.1 | HEART REPLACED BY TRANSPLANT |
| V42.6 | LUNG REPLACED BY TRANSPLANT |
| V42.7 | LIVER REPLACED BY TRANSPLANT |
| V42.81 | ORGAN OR TISSUE REPLACED BY TRANSPLANT, BONE MARROW |
| V42.82 | ORGAN OR TISSUE REPLACED BY TRANSPLANT, PERIPHERAL STEM CELLS |
| V42.83 | ORGAN OR TISSUE REPLACED BY TRANSPLANT, PANCREAS |
| V42.84 | ORGAN OR TISSUE REPLACED BY TRANSPLANT, INTESTINES |
| V42.89 | OTHER ORGAN OR TISSUE REPLACED BY TRANSPLANT |
| V42.9 | UNSPECIFIED ORGAN OR TISSUE REPLACED BY TRANSPLANT |
| V58.44 | AFTERCARE FOLLOWING ORGAN TRANSPLANT |
| Z48.298 | Encounter for aftercare following other organ transplant |
| Z94.0 | Kidney transplant status |
| Z94.1 | Heart transplant status |
| Z94.2 | Lung transplant status |
| Z94.4 | Liver transplant status |
| Z94.81 | Bone marrow transplant status |
| Z94.82 | Intestine transplant status |
| Z94.83 | Pancreas transplant status |
| Z94.84 | Stem cells transplant status |
| Z94.89 | Other transplanted organ and tissue status |
| Z94.9 | Transplanted organ and tissue status, unspecified |

**Supplement 1: Exclusion CPT Codes**

| **CPTCode** | **CPTName** | **CPTDescription** |
| --- | --- | --- |
| 50010 | RENAL EXPLORATION | RENAL EXPLORATION, NOT NECESSITATING OTHER SPECIFIC PROCEDURES |
| 50020 | DRG PERIRNL/RENAL ABSC OPEN | DRAINAGE OF PERIRENAL OR RENAL ABSCESS, OPEN |
| 50040 | NFROS NFROT W/DRG | NEPHROSTOMY, NEPHROTOMY WITH DRAINAGE |
| 50045 | NEPHROTOMY W/EXPLORATION | NEPHROTOMY, WITH EXPLORATION |
| 50060 | NL REMOVAL CALCULUS | NEPHROLITHOTOMY; REMOVAL OF CALCULUS |
| 50065 | NL SEC SURG OPERJ CALCULUS | NEPHROLITHOTOMY; SECONDARY SURGICAL OPERATION FOR CALCULUS |
| 50070 | NL COMP CGEN KDN ABNORMALITY | NEPHROLITHOTOMY; COMPLICATED BY CONGENITAL KIDNEY ABNORMALITY |
| 50075 | NL RMVL LG STAGHORN CALCULUS | NEPHROLITHOTOMY; REMOVAL OF LARGE STAGHORN CALCULUS FILLING RENAL PELVIS AND CALYCES (INCLUDING ANATROPHIC PYELOLITHOTOMY) |
| 50080 | PERQ NL/PL LITHOTRP SMPL<2CM | PERCUTANEOUS NEPHROLITHOTOMY OR PYELOLITHOTOMY, LITHOTRIPSY, STONE EXTRACTION, ANTEGRADE URETEROSCOPY, ANTEGRADE STENT PLACEMENT AND NEPHROSTOMY TUBE PLACEMENT, WHEN PERFORMED, INCLUDING IMAGING GUIDANCE; SIMPLE (EG, STONE[S] UP TO 2 CM IN SINGLE LOCATION OF KIDNEY OR RENAL PELVIS, NONBRANCHING STONES) |
| 50081 | PERQ NL/PL LITHOTRP CPLX>2CM | PERCUTANEOUS NEPHROLITHOTOMY OR PYELOLITHOTOMY, LITHOTRIPSY, STONE EXTRACTION, ANTEGRADE URETEROSCOPY, ANTEGRADE STENT PLACEMENT AND NEPHROSTOMY TUBE PLACEMENT, WHEN PERFORMED, INCLUDING IMAGING GUIDANCE; COMPLEX (EG, STONE[S] > 2 CM, BRANCHING STONES, STONES IN MULTIPLE LOCATIONS, URETER STONES, COMPLICATED ANATOMY) |
| 50100 | TRNSXJ/REPOS ABRRNT RNL VSLS | TRANSECTION OR REPOSITIONING OF ABERRANT RENAL VESSELS (SEPARATE PROCEDURE) |
| 50120 | PYELOTOMY W/EXPLORATION | PYELOTOMY; WITH EXPLORATION |
| 50125 | PYELOTOMY W/DRG PYELOSTOMY | PYELOTOMY; WITH DRAINAGE, PYELOSTOMY |
| 50130 | PYELOTOMY W/REMOVAL CALCULUS | PYELOTOMY; WITH REMOVAL OF CALCULUS (PYELOLITHOTOMY, PELVIOLITHOTOMY, INCLUDING COAGULUM PYELOLITHOTOMY) |
| 50135 | PYELOTOMY COMPLICATED | PYELOTOMY; COMPLICATED (EG, SECONDARY OPERATION, CONGENITAL KIDNEY ABNORMALITY) |
| 50205 | RENAL BX SURG EXPOSURE KDN | RENAL BIOPSY; BY SURGICAL EXPOSURE OF KIDNEY |
| 50220 | REMOVE KIDNEY OPEN | NEPHRECTOMY, INCLUDING PARTIAL URETERECTOMY, ANY OPEN APPROACH INCLUDING RIB RESECTION; |
| 50225 | REMOVAL KIDNEY OPEN COMPLEX | NEPHRECTOMY, INCLUDING PARTIAL URETERECTOMY, ANY OPEN APPROACH INCLUDING RIB RESECTION; COMPLICATED BECAUSE OF PREVIOUS SURGERY ON SAME KIDNEY |
| 50230 | REMOVAL KIDNEY OPEN RADICAL | NEPHRECTOMY, INCLUDING PARTIAL URETERECTOMY, ANY OPEN APPROACH INCLUDING RIB RESECTION; RADICAL, WITH REGIONAL LYMPHADENECTOMY AND/OR VENA CAVAL THROMBECTOMY |
| 50234 | REMOVAL OF KIDNEY & URETER | NEPHRECTOMY WITH TOTAL URETERECTOMY AND BLADDER CUFF; THROUGH SAME INCISION |
| 50236 | REMOVAL OF KIDNEY & URETER | NEPHRECTOMY WITH TOTAL URETERECTOMY AND BLADDER CUFF; THROUGH SEPARATE INCISION |
| 50240 | NEPHRECTOMY PARTIAL | NEPHRECTOMY, PARTIAL |
| 50250 | OPN ABLTJ 1/> RNL MAS CRYSRG | ABLATION, OPEN, 1 OR MORE RENAL MASS LESION(S), CRYOSURGICAL, INCLUDING INTRAOPERATIVE ULTRASOUND GUIDANCE AND MONITORING, IF PERFORMED |
| 50280 | EXC/UNROOFING CYST KIDNEY | EXCISION OR UNROOFING OF CYST(S) OF KIDNEY |
| 50290 | EXCISION PERINEPHRIC CYST | EXCISION OF PERINEPHRIC CYST |
| 50320 | REMOVE KIDNEY LIVING DONOR | DONOR NEPHRECTOMY (INCLUDING COLD PRESERVATION); OPEN, FROM LIVING DONOR |
| 50340 | RECIPIENT NEPHRECTOMY | RECIPIENT NEPHRECTOMY (SEPARATE PROCEDURE) |
| 50360 | RNL ALTRNSPLJ W/O RCP NFRCT | RENAL ALLOTRANSPLANTATION, IMPLANTATION OF GRAFT; WITHOUT RECIPIENT NEPHRECTOMY |
| 50365 | RNL ALTRNSPLJ W/RCP NFRCT | RENAL ALLOTRANSPLANTATION, IMPLANTATION OF GRAFT; WITH RECIPIENT NEPHRECTOMY |
| 50370 | RMVL TRANSPLANTED RNL ALGRFT | REMOVAL OF TRANSPLANTED RENAL ALLOGRAFT |
| 50380 | RNL AUTOTRNSPLJ RIMPLTJ KDN | RENAL AUTOTRANSPLANTATION, REIMPLANTATION OF KIDNEY |
| 50400 | REVISION OF KIDNEY/URETER | PYELOPLASTY (FOLEY Y-PYELOPLASTY), PLASTIC OPERATION ON RENAL PELVIS, WITH OR WITHOUT PLASTIC OPERATION ON URETER, NEPHROPEXY, NEPHROSTOMY, PYELOSTOMY, OR URETERAL SPLINTING; SIMPLE |
| 50405 | REVISION OF KIDNEY/URETER | PYELOPLASTY (FOLEY Y-PYELOPLASTY), PLASTIC OPERATION ON RENAL PELVIS, WITH OR WITHOUT PLASTIC OPERATION ON URETER, NEPHROPEXY, NEPHROSTOMY, PYELOSTOMY, OR URETERAL SPLINTING; COMPLICATED (CONGENITAL KIDNEY ABNORMALITY, SECONDARY PYELOPLASTY, SOLITARY KIDNEY, CALYCOPLASTY) |
| 50500 | REPAIR OF KIDNEY WOUND | NEPHRORRHAPHY, SUTURE OF KIDNEY WOUND OR INJURY |
| 50520 | CLOSE KIDNEY-SKIN FISTULA | CLOSURE OF NEPHROCUTANEOUS OR PYELOCUTANEOUS FISTULA |
| 50525 | CLOSE NEPHROVISCERAL FISTULA | CLOSURE OF NEPHROVISCERAL FISTULA (EG, RENOCOLIC), INCLUDING VISCERAL REPAIR; ABDOMINAL APPROACH |
| 50526 | CLOSE NEPHROVISCERAL FISTULA | CLOSURE OF NEPHROVISCERAL FISTULA (EG, RENOCOLIC), INCLUDING VISCERAL REPAIR; THORACIC APPROACH |
| 50540 | REVISION OF HORSESHOE KIDNEY | SYMPHYSIOTOMY FOR HORSESHOE KIDNEY WITH OR WITHOUT PYELOPLASTY AND/OR OTHER PLASTIC PROCEDURE, UNILATERAL OR BILATERAL (1 OPERATION) |
| 50541 | LAPARO ABLATE RENAL CYST | LAPAROSCOPY, SURGICAL; ABLATION OF RENAL CYSTS |
| 50542 | LAPARO ABLATE RENAL MASS | LAPAROSCOPY, SURGICAL; ABLATION OF RENAL MASS LESION(S), INCLUDING INTRAOPERATIVE ULTRASOUND GUIDANCE AND MONITORING, WHEN PERFORMED |
| 50543 | LAPARO PARTIAL NEPHRECTOMY | LAPAROSCOPY, SURGICAL; PARTIAL NEPHRECTOMY |
| 50544 | LAPAROSCOPY PYELOPLASTY | LAPAROSCOPY, SURGICAL; PYELOPLASTY |
| 50545 | LAPARO RADICAL NEPHRECTOMY | LAPAROSCOPY, SURGICAL; RADICAL NEPHRECTOMY (INCLUDES REMOVAL OF GEROTA'S FASCIA AND SURROUNDING FATTY TISSUE, REMOVAL OF REGIONAL LYMPH NODES, AND ADRENALECTOMY) |
| 50546 | LAPAROSCOPIC NEPHRECTOMY | LAPAROSCOPY, SURGICAL; NEPHRECTOMY, INCLUDING PARTIAL URETERECTOMY |
| 50547 | LAPARO REMOVAL DONOR KIDNEY | LAPAROSCOPY, SURGICAL; DONOR NEPHRECTOMY (INCLUDING COLD PRESERVATION), FROM LIVING DONOR |
| 50548 | LAPARO REMOVE W/URETER | LAPAROSCOPY, SURGICAL; NEPHRECTOMY WITH TOTAL URETERECTOMY |
| 50562 | RENAL SCOPE W/TUMOR RESECT | RENAL ENDOSCOPY THROUGH ESTABLISHED NEPHROSTOMY OR PYELOSTOMY, WITH OR WITHOUT IRRIGATION, INSTILLATION, OR URETEROPYELOGRAPHY, EXCLUSIVE OF RADIOLOGIC SERVICE; WITH RESECTION OF TUMOR |
| 50590 | FRAGMENTING OF KIDNEY STONE | LITHOTRIPSY, EXTRACORPOREAL SHOCK WAVE |
| 50600 | EXPLORATION OF URETER | URETEROTOMY WITH EXPLORATION OR DRAINAGE (SEPARATE PROCEDURE) |
| 50605 | INSERT URETERAL SUPPORT | URETEROTOMY FOR INSERTION OF INDWELLING STENT, ALL TYPES |
| 50610 | REMOVAL OF URETER STONE | URETEROLITHOTOMY; UPPER ONE-THIRD OF URETER |
| 50620 | REMOVAL OF URETER STONE | URETEROLITHOTOMY; MIDDLE ONE-THIRD OF URETER |
| 50630 | REMOVAL OF URETER STONE | URETEROLITHOTOMY; LOWER ONE-THIRD OF URETER |
| 50650 | REMOVAL OF URETER | URETERECTOMY, WITH BLADDER CUFF (SEPARATE PROCEDURE) |
| 50660 | REMOVAL OF URETER | URETERECTOMY, TOTAL, ECTOPIC URETER, COMBINATION ABDOMINAL, VAGINAL AND/OR PERINEAL APPROACH |
| 50700 | REVISION OF URETER | URETEROPLASTY, PLASTIC OPERATION ON URETER (EG, STRICTURE) |
| 50715 | RELEASE OF URETER | URETEROLYSIS, WITH OR WITHOUT REPOSITIONING OF URETER FOR RETROPERITONEAL FIBROSIS |
| 50722 | RELEASE OF URETER | URETEROLYSIS FOR OVARIAN VEIN SYNDROME |
| 50725 | RELEASE/REVISE URETER | URETEROLYSIS FOR RETROCAVAL URETER, WITH REANASTOMOSIS OF UPPER URINARY TRACT OR VENA CAVA |
| 50727 | REVISE URETER | REVISION OF URINARY-CUTANEOUS ANASTOMOSIS (ANY TYPE UROSTOMY); |
| 50728 | REVISE URETER | REVISION OF URINARY-CUTANEOUS ANASTOMOSIS (ANY TYPE UROSTOMY); WITH REPAIR OF FASCIAL DEFECT AND HERNIA |
| 50740 | FUSION OF URETER & KIDNEY | URETEROPYELOSTOMY, ANASTOMOSIS OF URETER AND RENAL PELVIS |
| 50750 | FUSION OF URETER & KIDNEY | URETEROCALYCOSTOMY, ANASTOMOSIS OF URETER TO RENAL CALYX |
| 50760 | URETEROURETEROSTOMY | URETEROURETEROSTOMY |
| 50770 | SPLICING OF URETERS | TRANSURETEROURETEROSTOMY, ANASTOMOSIS OF URETER TO CONTRALATERAL URETER |
| 50780 | REIMPLANT URETER IN BLADDER | URETERONEOCYSTOSTOMY; ANASTOMOSIS OF SINGLE URETER TO BLADDER |
| 50782 | REIMPLANT URETER IN BLADDER | URETERONEOCYSTOSTOMY; ANASTOMOSIS OF DUPLICATED URETER TO BLADDER |
| 50783 | REIMPLANT URETER IN BLADDER | URETERONEOCYSTOSTOMY; WITH EXTENSIVE URETERAL TAILORING |
| 50785 | REIMPLANT URETER IN BLADDER | URETERONEOCYSTOSTOMY; WITH VESICO-PSOAS HITCH OR BLADDER FLAP |
| 50800 | IMPLANT URETER IN BOWEL | URETEROENTEROSTOMY, DIRECT ANASTOMOSIS OF URETER TO INTESTINE |
| 50810 | FUSION OF URETER & BOWEL | URETEROSIGMOIDOSTOMY, WITH CREATION OF SIGMOID BLADDER AND ESTABLISHMENT OF ABDOMINAL OR PERINEAL COLOSTOMY, INCLUDING INTESTINE ANASTOMOSIS |
| 50815 | URINE SHUNT TO INTESTINE | URETEROCOLON CONDUIT, INCLUDING INTESTINE ANASTOMOSIS |
| 50820 | CONSTRUCT BOWEL BLADDER | URETEROILEAL CONDUIT (ILEAL BLADDER), INCLUDING INTESTINE ANASTOMOSIS (BRICKER OPERATION) |
| 50825 | CONSTRUCT BOWEL BLADDER | CONTINENT DIVERSION, INCLUDING INTESTINE ANASTOMOSIS USING ANY SEGMENT OF SMALL AND/OR LARGE INTESTINE (KOCK POUCH OR CAMEY ENTEROCYSTOPLASTY) |
| 50830 | REVISE URINE FLOW | URINARY UNDIVERSION (EG, TAKING DOWN OF URETEROILEAL CONDUIT, URETEROSIGMOIDOSTOMY OR URETEROENTEROSTOMY WITH URETEROURETEROSTOMY OR URETERONEOCYSTOSTOMY) |
| 50840 | REPLACE URETER BY BOWEL | REPLACEMENT OF ALL OR PART OF URETER BY INTESTINE SEGMENT, INCLUDING INTESTINE ANASTOMOSIS |
| 50845 | APPENDICO-VESICOSTOMY | CUTANEOUS APPENDICO-VESICOSTOMY |
| 50860 | TRANSPLANT URETER TO SKIN | URETEROSTOMY, TRANSPLANTATION OF URETER TO SKIN |
| 50900 | REPAIR OF URETER | URETERORRHAPHY, SUTURE OF URETER (SEPARATE PROCEDURE) |
| 50920 | CLOSURE URETER/SKIN FISTULA | CLOSURE OF URETEROCUTANEOUS FISTULA |
| 50930 | CLOSURE URETER/BOWEL FISTULA | CLOSURE OF URETEROVISCERAL FISTULA (INCLUDING VISCERAL REPAIR) |
| 50940 | RELEASE OF URETER | DELIGATION OF URETER |
| 50945 | LAPAROSCOPY URETEROLITHOTOMY | LAPAROSCOPY, SURGICAL; URETEROLITHOTOMY |
| 50947 | LAPARO NEW URETER/BLADDER | LAPAROSCOPY, SURGICAL; URETERONEOCYSTOSTOMY WITH CYSTOSCOPY AND URETERAL STENT PLACEMENT |
| 50948 | LAPARO NEW URETER/BLADDER | LAPAROSCOPY, SURGICAL; URETERONEOCYSTOSTOMY WITHOUT CYSTOSCOPY AND URETERAL STENT PLACEMENT |
| 51020 | CYSTOTOMY/CYSTOSTOMY W/FULG | CYSTOTOMY OR CYSTOSTOMY, WITH FULGURATION AND/OR INSERTION OF RADIOACTIVE MATERIAL |
| 51030 | INCISE & TREAT BLADDER | CYSTOTOMY OR CYSTOSTOMY; WITH CRYOSURGICAL DESTRUCTION OF INTRAVESICAL LESION |
| 51040 | INCISE & DRAIN BLADDER | CYSTOSTOMY, CYSTOTOMY WITH DRAINAGE |
| 51045 | INCISE BLADDER/DRAIN URETER | CYSTOTOMY, WITH INSERTION OF URETERAL CATHETER OR STENT (SEPARATE PROCEDURE) |
| 51050 | REMOVAL OF BLADDER STONE | CYSTOLITHOTOMY, CYSTOTOMY WITH REMOVAL OF CALCULUS, WITHOUT VESICAL NECK RESECTION |
| 51060 | REMOVAL OF URETER STONE | TRANSVESICAL URETEROLITHOTOMY |
| 51065 | REMOVE URETER CALCULUS | CYSTOTOMY, WITH CALCULUS BASKET EXTRACTION AND/OR ULTRASONIC OR ELECTROHYDRAULIC FRAGMENTATION OF URETERAL CALCULUS |
| 51080 | DRAINAGE OF BLADDER ABSCESS | DRAINAGE OF PERIVESICAL OR PREVESICAL SPACE ABSCESS |
| 51500 | REMOVAL OF BLADDER CYST | EXCISION OF URACHAL CYST OR SINUS, WITH OR WITHOUT UMBILICAL HERNIA REPAIR |
| 51520 | REMOVAL OF BLADDER LESION | CYSTOTOMY; FOR SIMPLE EXCISION OF VESICAL NECK (SEPARATE PROCEDURE) |
| 51525 | REMOVAL OF BLADDER LESION | CYSTOTOMY; FOR EXCISION OF BLADDER DIVERTICULUM, SINGLE OR MULTIPLE (SEPARATE PROCEDURE) |
| 51530 | REMOVAL OF BLADDER LESION | CYSTOTOMY; FOR EXCISION OF BLADDER TUMOR |
| 51535 | REPAIR OF URETER LESION | CYSTOTOMY FOR EXCISION, INCISION, OR REPAIR OF URETEROCELE |
| 51550 | PARTIAL REMOVAL OF BLADDER | CYSTECTOMY, PARTIAL; SIMPLE |
| 51555 | PARTIAL REMOVAL OF BLADDER | CYSTECTOMY, PARTIAL; COMPLICATED (EG, POSTRADIATION, PREVIOUS SURGERY, DIFFICULT LOCATION) |
| 51565 | REVISE BLADDER & URETER(S) | CYSTECTOMY, PARTIAL, WITH REIMPLANTATION OF URETER(S) INTO BLADDER (URETERONEOCYSTOSTOMY) |
| 51570 | REMOVAL OF BLADDER | CYSTECTOMY, COMPLETE; (SEPARATE PROCEDURE) |
| 51575 | REMOVAL OF BLADDER & NODES | CYSTECTOMY, COMPLETE; WITH BILATERAL PELVIC LYMPHADENECTOMY, INCLUDING EXTERNAL ILIAC, HYPOGASTRIC, AND OBTURATOR NODES |
| 51580 | REMOVE BLADDER/REVISE TRACT | CYSTECTOMY, COMPLETE, WITH URETEROSIGMOIDOSTOMY OR URETEROCUTANEOUS TRANSPLANTATIONS; |
| 51585 | REMOVAL OF BLADDER & NODES | CYSTECTOMY, COMPLETE, WITH URETEROSIGMOIDOSTOMY OR URETEROCUTANEOUS TRANSPLANTATIONS; WITH BILATERAL PELVIC LYMPHADENECTOMY, INCLUDING EXTERNAL ILIAC, HYPOGASTRIC, AND OBTURATOR NODES |
| 51590 | REMOVE BLADDER/REVISE TRACT | CYSTECTOMY, COMPLETE, WITH URETEROILEAL CONDUIT OR SIGMOID BLADDER, INCLUDING INTESTINE ANASTOMOSIS; |
| 51595 | REMOVE BLADDER/REVISE TRACT | CYSTECTOMY, COMPLETE, WITH URETEROILEAL CONDUIT OR SIGMOID BLADDER, INCLUDING INTESTINE ANASTOMOSIS; WITH BILATERAL PELVIC LYMPHADENECTOMY, INCLUDING EXTERNAL ILIAC, HYPOGASTRIC, AND OBTURATOR NODES |
| 51596 | REMOVE BLADDER/CREATE POUCH | CYSTECTOMY, COMPLETE, WITH CONTINENT DIVERSION, ANY OPEN TECHNIQUE, USING ANY SEGMENT OF SMALL AND/OR LARGE INTESTINE TO CONSTRUCT NEOBLADDER |
| 51597 | REMOVAL OF PELVIC STRUCTURES | PELVIC EXENTERATION, COMPLETE, FOR VESICAL, PROSTATIC OR URETHRAL MALIGNANCY, WITH REMOVAL OF BLADDER AND URETERAL TRANSPLANTATIONS, WITH OR WITHOUT HYSTERECTOMY AND/OR ABDOMINOPERINEAL RESECTION OF RECTUM AND COLON AND COLOSTOMY, OR ANY COMBINATION THEREOF |
| 51800 | REVISION OF BLADDER/URETHRA | CYSTOPLASTY OR CYSTOURETHROPLASTY, PLASTIC OPERATION ON BLADDER AND/OR VESICAL NECK (ANTERIOR Y-PLASTY, VESICAL FUNDUS RESECTION), ANY PROCEDURE, WITH OR WITHOUT WEDGE RESECTION OF POSTERIOR VESICAL NECK |
| 51820 | REVISION OF URINARY TRACT | CYSTOURETHROPLASTY WITH UNILATERAL OR BILATERAL URETERONEOCYSTOSTOMY |
| 51840 | ATTACH BLADDER/URETHRA | ANTERIOR VESICOURETHROPEXY, OR URETHROPEXY (EG, MARSHALL-MARCHETTI-KRANTZ, BURCH); SIMPLE |
| 51841 | ATTACH BLADDER/URETHRA | ANTERIOR VESICOURETHROPEXY, OR URETHROPEXY (EG, MARSHALL-MARCHETTI-KRANTZ, BURCH); COMPLICATED (EG, SECONDARY REPAIR) |
| 51845 | REPAIR BLADDER NECK | ABDOMINO-VAGINAL VESICAL NECK SUSPENSION, WITH OR WITHOUT ENDOSCOPIC CONTROL (EG, STAMEY, RAZ, MODIFIED PEREYRA) |
| 51860 | REPAIR OF BLADDER WOUND | CYSTORRHAPHY, SUTURE OF BLADDER WOUND, INJURY OR RUPTURE; SIMPLE |
| 51865 | REPAIR OF BLADDER WOUND | CYSTORRHAPHY, SUTURE OF BLADDER WOUND, INJURY OR RUPTURE; COMPLICATED |
| 51880 | REPAIR OF BLADDER OPENING | CLOSURE OF CYSTOSTOMY (SEPARATE PROCEDURE) |
| 51900 | REPAIR BLADDER/VAGINA LESION | CLOSURE OF VESICOVAGINAL FISTULA, ABDOMINAL APPROACH |
| 51920 | CLOSE BLADDER-UTERUS FISTULA | CLOSURE OF VESICOUTERINE FISTULA; |
| 51925 | HYSTERECTOMY/BLADDER REPAIR | CLOSURE OF VESICOUTERINE FISTULA; WITH HYSTERECTOMY |
| 51940 | CORRECTION OF BLADDER DEFECT | CLOSURE, EXSTROPHY OF BLADDER |
| 51960 | REVISION OF BLADDER & BOWEL | ENTEROCYSTOPLASTY, INCLUDING INTESTINAL ANASTOMOSIS |
| 51980 | CONSTRUCT BLADDER OPENING | CUTANEOUS VESICOSTOMY |
| 51990 | LAPARO URETHRAL SUSPENSION | LAPAROSCOPY, SURGICAL; URETHRAL SUSPENSION FOR STRESS INCONTINENCE |
| 51992 | LAPARO SLING OPERATION | LAPAROSCOPY, SURGICAL; SLING OPERATION FOR STRESS INCONTINENCE (EG, FASCIA OR SYNTHETIC) |
| 52400 | CYSTOURETERO W/CONGEN REPR | CYSTOURETHROSCOPY WITH INCISION, FULGURATION, OR RESECTION OF CONGENITAL POSTERIOR URETHRAL VALVES, OR CONGENITAL OBSTRUCTIVE HYPERTROPHIC MUCOSAL FOLDS |
| 52450 | INCISION OF PROSTATE | TRANSURETHRAL INCISION OF PROSTATE |
| 52500 | REVISION OF BLADDER NECK | TRANSURETHRAL RESECTION OF BLADDER NECK (SEPARATE PROCEDURE) |
| 52601 | PROSTATECTOMY (TURP) | TRANSURETHRAL ELECTROSURGICAL RESECTION OF PROSTATE, INCLUDING CONTROL OF POSTOPERATIVE BLEEDING, COMPLETE (VASECTOMY, MEATOTOMY, CYSTOURETHROSCOPY, URETHRAL CALIBRATION AND/OR DILATION, AND INTERNAL URETHROTOMY ARE INCLUDED) |
| 52630 | REMOVE PROSTATE REGROWTH | TRANSURETHRAL RESECTION; RESIDUAL OR REGROWTH OF OBSTRUCTIVE PROSTATE TISSUE INCLUDING CONTROL OF POSTOPERATIVE BLEEDING, COMPLETE (VASECTOMY, MEATOTOMY, CYSTOURETHROSCOPY, URETHRAL CALIBRATION AND/OR DILATION, AND INTERNAL URETHROTOMY ARE INCLUDED) |
| 52640 | RELIEVE BLADDER CONTRACTURE | TRANSURETHRAL RESECTION; OF POSTOPERATIVE BLADDER NECK CONTRACTURE |
| 52647 | LASER SURGERY OF PROSTATE | LASER COAGULATION OF PROSTATE, INCLUDING CONTROL OF POSTOPERATIVE BLEEDING, COMPLETE (VASECTOMY, MEATOTOMY, CYSTOURETHROSCOPY, URETHRAL CALIBRATION AND/OR DILATION, AND INTERNAL URETHROTOMY ARE INCLUDED IF PERFORMED) |
| 52648 | LASER SURGERY OF PROSTATE | LASER VAPORIZATION OF PROSTATE, INCLUDING CONTROL OF POSTOPERATIVE BLEEDING, COMPLETE (VASECTOMY, MEATOTOMY, CYSTOURETHROSCOPY, URETHRAL CALIBRATION AND/OR DILATION, INTERNAL URETHROTOMY AND TRANSURETHRAL RESECTION OF PROSTATE ARE INCLUDED IF PERFORMED) |
| 52649 | PROSTATE LASER ENUCLEATION | LASER ENUCLEATION OF THE PROSTATE WITH MORCELLATION, INCLUDING CONTROL OF POSTOPERATIVE BLEEDING, COMPLETE (VASECTOMY, MEATOTOMY, CYSTOURETHROSCOPY, URETHRAL CALIBRATION AND/OR DILATION, INTERNAL URETHROTOMY AND TRANSURETHRAL RESECTION OF PROSTATE ARE INCLUDED IF PERFORMED) |
| 52700 | DRAINAGE OF PROSTATE ABSCESS | TRANSURETHRAL DRAINAGE OF PROSTATIC ABSCESS |
| 53010 | INCISION OF URETHRA | URETHROTOMY OR URETHROSTOMY, EXTERNAL (SEPARATE PROCEDURE); PERINEAL URETHRA, EXTERNAL |
| 53040 | DRAINAGE OF URETHRA ABSCESS | DRAINAGE OF DEEP PERIURETHRAL ABSCESS |
| 53080 | DRAINAGE OF URINARY LEAKAGE | DRAINAGE OF PERINEAL URINARY EXTRAVASATION; UNCOMPLICATED (SEPARATE PROCEDURE) |
| 53085 | DRAINAGE OF URINARY LEAKAGE | DRAINAGE OF PERINEAL URINARY EXTRAVASATION; COMPLICATED |
| 53210 | REMOVAL OF URETHRA | URETHRECTOMY, TOTAL, INCLUDING CYSTOSTOMY; FEMALE |
| 53215 | REMOVAL OF URETHRA | URETHRECTOMY, TOTAL, INCLUDING CYSTOSTOMY; MALE |
| 53220 | TREATMENT OF URETHRA LESION | EXCISION OR FULGURATION OF CARCINOMA OF URETHRA |
| 53230 | REMOVAL OF URETHRA LESION | EXCISION OF URETHRAL DIVERTICULUM (SEPARATE PROCEDURE); FEMALE |
| 53235 | REMOVAL OF URETHRA LESION | EXCISION OF URETHRAL DIVERTICULUM (SEPARATE PROCEDURE); MALE |
| 53240 | SURGERY FOR URETHRA POUCH | MARSUPIALIZATION OF URETHRAL DIVERTICULUM, MALE OR FEMALE |
| 53250 | REMOVAL OF URETHRA GLAND | EXCISION OF BULBOURETHRAL GLAND (COWPER'S GLAND) |
| 53400 | REVISE URETHRA STAGE 1 | URETHROPLASTY; FIRST STAGE, FOR FISTULA, DIVERTICULUM, OR STRICTURE (EG, JOHANNSEN TYPE) |
| 53405 | REVISE URETHRA STAGE 2 | URETHROPLASTY; SECOND STAGE (FORMATION OF URETHRA), INCLUDING URINARY DIVERSION |
| 53410 | RECONSTRUCTION OF URETHRA | URETHROPLASTY, 1-STAGE RECONSTRUCTION OF MALE ANTERIOR URETHRA |
| 53415 | RECONSTRUCTION OF URETHRA | URETHROPLASTY, TRANSPUBIC OR PERINEAL, 1-STAGE, FOR RECONSTRUCTION OR REPAIR OF PROSTATIC OR MEMBRANOUS URETHRA |
| 53420 | RECONSTRUCT URETHRA STAGE 1 | URETHROPLASTY, 2-STAGE RECONSTRUCTION OR REPAIR OF PROSTATIC OR MEMBRANOUS URETHRA; FIRST STAGE |
| 53425 | RECONSTRUCT URETHRA STAGE 2 | URETHROPLASTY, 2-STAGE RECONSTRUCTION OR REPAIR OF PROSTATIC OR MEMBRANOUS URETHRA; SECOND STAGE |
| 53431 | RECONSTRUCT URETHRA/BLADDER | URETHROPLASTY WITH TUBULARIZATION OF POSTERIOR URETHRA AND/OR LOWER BLADDER FOR INCONTINENCE (EG, TENAGO, LEADBETTER PROCEDURE) |
| 53440 | MALE SLING PROCEDURE | SLING OPERATION FOR CORRECTION OF MALE URINARY INCONTINENCE (EG, FASCIA OR SYNTHETIC) |
| 53442 | REMOVE/REVISE MALE SLING | REMOVAL OR REVISION OF SLING FOR MALE URINARY INCONTINENCE (EG, FASCIA OR SYNTHETIC) |
| 53444 | INSERT TANDEM CUFF | INSERTION OF TANDEM CUFF (DUAL CUFF) |
| 53445 | INSERT URO/VES NCK SPHINCTER | INSERTION OF INFLATABLE URETHRAL/BLADDER NECK SPHINCTER, INCLUDING PLACEMENT OF PUMP, RESERVOIR, AND CUFF |
| 53446 | REMOVE URO SPHINCTER | REMOVAL OF INFLATABLE URETHRAL/BLADDER NECK SPHINCTER, INCLUDING PUMP, RESERVOIR, AND CUFF |
| 53447 | REMOVE/REPLACE UR SPHINCTER | REMOVAL AND REPLACEMENT OF INFLATABLE URETHRAL/BLADDER NECK SPHINCTER INCLUDING PUMP, RESERVOIR, AND CUFF AT THE SAME OPERATIVE SESSION |
| 53449 | REPAIR URO SPHINCTER | REPAIR OF INFLATABLE URETHRAL/BLADDER NECK SPHINCTER, INCLUDING PUMP, RESERVOIR, AND CUFF |
| 53450 | REVISION OF URETHRA | URETHROMEATOPLASTY, WITH MUCOSAL ADVANCEMENT |
| 53460 | REVISION OF URETHRA | URETHROMEATOPLASTY, WITH PARTIAL EXCISION OF DISTAL URETHRAL SEGMENT (RICHARDSON TYPE PROCEDURE) |
| 53500 | URETHRLYS TRANSVAG W/ SCOPE | URETHROLYSIS, TRANSVAGINAL, SECONDARY, OPEN, INCLUDING CYSTOURETHROSCOPY (EG, POSTSURGICAL OBSTRUCTION, SCARRING) |
| 53502 | REPAIR OF URETHRA INJURY | URETHRORRHAPHY, SUTURE OF URETHRAL WOUND OR INJURY, FEMALE |
| 53505 | REPAIR OF URETHRA INJURY | URETHRORRHAPHY, SUTURE OF URETHRAL WOUND OR INJURY; PENILE |
| 53510 | REPAIR OF URETHRA INJURY | URETHRORRHAPHY, SUTURE OF URETHRAL WOUND OR INJURY; PERINEAL |
| 53515 | REPAIR OF URETHRA INJURY | URETHRORRHAPHY, SUTURE OF URETHRAL WOUND OR INJURY; PROSTATOMEMBRANOUS |
| 53520 | REPAIR OF URETHRA DEFECT | CLOSURE OF URETHROSTOMY OR URETHROCUTANEOUS FISTULA, MALE (SEPARATE PROCEDURE) |
| 53850 | PROSTATIC MICROWAVE THERMOTX | TRANSURETHRAL DESTRUCTION OF PROSTATE TISSUE; BY MICROWAVE THERMOTHERAPY |
| 53852 | PROSTATIC RF THERMOTX | TRANSURETHRAL DESTRUCTION OF PROSTATE TISSUE; BY RADIOFREQUENCY THERMOTHERAPY |
| 53860 | TRANSURETHRAL RF TREATMENT | TRANSURETHRAL RADIOFREQUENCY MICRO-REMODELING OF THE FEMALE BLADDER NECK AND PROXIMAL URETHRA FOR STRESS URINARY INCONTINENCE |
| 54110 | TREATMENT OF PENIS LESION | EXCISION OF PENILE PLAQUE (PEYRONIE DISEASE); |
| 54111 | TREAT PENIS LESION GRAFT | EXCISION OF PENILE PLAQUE (PEYRONIE DISEASE); WITH GRAFT TO 5 CM IN LENGTH |
| 54112 | TREAT PENIS LESION GRAFT | EXCISION OF PENILE PLAQUE (PEYRONIE DISEASE); WITH GRAFT GREATER THAN 5 CM IN LENGTH |
| 54115 | TREATMENT OF PENIS LESION | REMOVAL FOREIGN BODY FROM DEEP PENILE TISSUE (EG, PLASTIC IMPLANT) |
| 54120 | PARTIAL REMOVAL OF PENIS | AMPUTATION OF PENIS; PARTIAL |
| 54125 | REMOVAL OF PENIS | AMPUTATION OF PENIS; COMPLETE |
| 54130 | REMOVE PENIS & NODES | AMPUTATION OF PENIS, RADICAL; WITH BILATERAL INGUINOFEMORAL LYMPHADENECTOMY |
| 54135 | REMOVE PENIS & NODES | AMPUTATION OF PENIS, RADICAL; IN CONTINUITY WITH BILATERAL PELVIC LYMPHADENECTOMY, INCLUDING EXTERNAL ILIAC, HYPOGASTRIC AND OBTURATOR NODES |
| 54205 | NJX PX PEYRONIE DS EXPS PLAQ | INJECTION PROCEDURE FOR PEYRONIE DISEASE; WITH SURGICAL EXPOSURE OF PLAQUE |
| 54300 | REVISION OF PENIS | PLASTIC OPERATION OF PENIS FOR STRAIGHTENING OF CHORDEE (EG, HYPOSPADIAS), WITH OR WITHOUT MOBILIZATION OF URETHRA |
| 54304 | REVISION OF PENIS | PLASTIC OPERATION ON PENIS FOR CORRECTION OF CHORDEE OR FOR FIRST STAGE HYPOSPADIAS REPAIR WITH OR WITHOUT TRANSPLANTATION OF PREPUCE AND/OR SKIN FLAPS |
| 54308 | RECONSTRUCTION OF URETHRA | URETHROPLASTY FOR SECOND STAGE HYPOSPADIAS REPAIR (INCLUDING URINARY DIVERSION); LESS THAN 3 CM |
| 54312 | RECONSTRUCTION OF URETHRA | URETHROPLASTY FOR SECOND STAGE HYPOSPADIAS REPAIR (INCLUDING URINARY DIVERSION); GREATER THAN 3 CM |
| 54316 | RECONSTRUCTION OF URETHRA | URETHROPLASTY FOR SECOND STAGE HYPOSPADIAS REPAIR (INCLUDING URINARY DIVERSION) WITH FREE SKIN GRAFT OBTAINED FROM SITE OTHER THAN GENITALIA |
| 54318 | RECONSTRUCTION OF URETHRA | URETHROPLASTY FOR THIRD STAGE HYPOSPADIAS REPAIR TO RELEASE PENIS FROM SCROTUM (EG, THIRD STAGE CECIL REPAIR) |
| 54322 | RECONSTRUCTION OF URETHRA | 1-STAGE DISTAL HYPOSPADIAS REPAIR (WITH OR WITHOUT CHORDEE OR CIRCUMCISION); WITH SIMPLE MEATAL ADVANCEMENT (EG, MAGPI, V-FLAP) |
| 54324 | RECONSTRUCTION OF URETHRA | 1-STAGE DISTAL HYPOSPADIAS REPAIR (WITH OR WITHOUT CHORDEE OR CIRCUMCISION); WITH URETHROPLASTY BY LOCAL SKIN FLAPS (EG, FLIP-FLAP, PREPUCIAL FLAP) |
| 54326 | RECONSTRUCTION OF URETHRA | 1-STAGE DISTAL HYPOSPADIAS REPAIR (WITH OR WITHOUT CHORDEE OR CIRCUMCISION); WITH URETHROPLASTY BY LOCAL SKIN FLAPS AND MOBILIZATION OF URETHRA |
| 54328 | REVISE PENIS/URETHRA | 1-STAGE DISTAL HYPOSPADIAS REPAIR (WITH OR WITHOUT CHORDEE OR CIRCUMCISION); WITH EXTENSIVE DISSECTION TO CORRECT CHORDEE AND URETHROPLASTY WITH LOCAL SKIN FLAPS, SKIN GRAFT PATCH, AND/OR ISLAND FLAP |
| 54332 | REVISE PENIS/URETHRA | 1-STAGE PROXIMAL PENILE OR PENOSCROTAL HYPOSPADIAS REPAIR REQUIRING EXTENSIVE DISSECTION TO CORRECT CHORDEE AND URETHROPLASTY BY USE OF SKIN GRAFT TUBE AND/OR ISLAND FLAP |
| 54336 | REVISE PENIS/URETHRA | 1-STAGE PERINEAL HYPOSPADIAS REPAIR REQUIRING EXTENSIVE DISSECTION TO CORRECT CHORDEE AND URETHROPLASTY BY USE OF SKIN GRAFT TUBE AND/OR ISLAND FLAP |
| 54340 | RPR HYPSPAD COMP SIMPLE | REPAIR OF HYPOSPADIAS COMPLICATION(S) (IE, FISTULA, STRICTURE, DIVERTICULA); BY CLOSURE, INCISION, OR EXCISION, SIMPLE |
| 54348 | RPR HYPSPAD COMP DSJ & URTP | REPAIR OF HYPOSPADIAS COMPLICATION(S) (IE, FISTULA, STRICTURE, DIVERTICULA); REQUIRING EXTENSIVE DISSECTION, AND URETHROPLASTY WITH FLAP, PATCH OR TUBED GRAFT (INCLUDING URINARY DIVERSION, WHEN PERFORMED) |
| 54352 | REVJ PRIOR HYPSPAD REPAIR | REVISION OF PRIOR HYPOSPADIAS REPAIR REQUIRING EXTENSIVE DISSECTION AND EXCISION OF PREVIOUSLY CONSTRUCTED STRUCTURES INCLUDING RE-RELEASE OF CHORDEE AND RECONSTRUCTION OF URETHRA AND PENIS BY USE OF LOCAL SKIN AS GRAFTS AND ISLAND FLAPS AND SKIN BROUGHT IN AS FLAPS OR GRAFTS |
| 54360 | PENIS PLASTIC SURGERY | PLASTIC OPERATION ON PENIS TO CORRECT ANGULATION |
| 54380 | REPAIR PENIS | PLASTIC OPERATION ON PENIS FOR EPISPADIAS DISTAL TO EXTERNAL SPHINCTER; |
| 54390 | REPAIR PENIS AND BLADDER | PLASTIC OPERATION ON PENIS FOR EPISPADIAS DISTAL TO EXTERNAL SPHINCTER; WITH EXSTROPHY OF BLADDER |
| 54400 | INSERT SEMI-RIGID PROSTHESIS | INSERTION OF PENILE PROSTHESIS; NON-INFLATABLE (SEMI-RIGID) |
| 54401 | INSERT SELF-CONTD PROSTHESIS | INSERTION OF PENILE PROSTHESIS; INFLATABLE (SELF-CONTAINED) |
| 54405 | INSERT MULTI-COMP PENIS PROS | INSERTION OF MULTI-COMPONENT, INFLATABLE PENILE PROSTHESIS, INCLUDING PLACEMENT OF PUMP, CYLINDERS, AND RESERVOIR |
| 54406 | REMOVE MUTI-COMP PENIS PROS | REMOVAL OF ALL COMPONENTS OF A MULTI-COMPONENT, INFLATABLE PENILE PROSTHESIS WITHOUT REPLACEMENT OF PROSTHESIS |
| 54408 | REPAIR MULTI-COMP PENIS PROS | REPAIR OF COMPONENT(S) OF A MULTI-COMPONENT, INFLATABLE PENILE PROSTHESIS |
| 54410 | REMOVE/REPLACE PENIS PROSTH | REMOVAL AND REPLACEMENT OF ALL COMPONENT(S) OF A MULTI-COMPONENT, INFLATABLE PENILE PROSTHESIS AT THE SAME OPERATIVE SESSION |
| 54411 | REMOV/REPLC PENIS PROS COMP | REMOVAL AND REPLACEMENT OF ALL COMPONENTS OF A MULTI-COMPONENT INFLATABLE PENILE PROSTHESIS THROUGH AN INFECTED FIELD AT THE SAME OPERATIVE SESSION, INCLUDING IRRIGATION AND DEBRIDEMENT OF INFECTED TISSUE |
| 54415 | REMOVE SELF-CONTD PENIS PROS | REMOVAL OF NON-INFLATABLE (SEMI-RIGID) OR INFLATABLE (SELF-CONTAINED) PENILE PROSTHESIS, WITHOUT REPLACEMENT OF PROSTHESIS |
| 54416 | REMV/REPL PENIS CONTAIN PROS | REMOVAL AND REPLACEMENT OF NON-INFLATABLE (SEMI-RIGID) OR INFLATABLE (SELF-CONTAINED) PENILE PROSTHESIS AT THE SAME OPERATIVE SESSION |
| 54417 | REMV/REPLC PENIS PROS COMPL | REMOVAL AND REPLACEMENT OF NON-INFLATABLE (SEMI-RIGID) OR INFLATABLE (SELF-CONTAINED) PENILE PROSTHESIS THROUGH AN INFECTED FIELD AT THE SAME OPERATIVE SESSION, INCLUDING IRRIGATION AND DEBRIDEMENT OF INFECTED TISSUE |
| 54420 | REVISION OF PENIS | CORPORA CAVERNOSA-SAPHENOUS VEIN SHUNT (PRIAPISM OPERATION), UNILATERAL OR BILATERAL |
| 54430 | REVISION OF PENIS | CORPORA CAVERNOSA-CORPUS SPONGIOSUM SHUNT (PRIAPISM OPERATION), UNILATERAL OR BILATERAL |
| 54435 | REVISION OF PENIS | CORPORA CAVERNOSA-GLANS PENIS FISTULIZATION (EG, BIOPSY NEEDLE, WINTER PROCEDURE, RONGEUR, OR PUNCH) FOR PRIAPISM |
| 54440 | REPAIR OF PENIS | PLASTIC OPERATION OF PENIS FOR INJURY |
| 54512 | EXCISE LESION TESTIS | EXCISION OF EXTRAPARENCHYMAL LESION OF TESTIS |
| 54520 | REMOVAL OF TESTIS | ORCHIECTOMY, SIMPLE (INCLUDING SUBCAPSULAR), WITH OR WITHOUT TESTICULAR PROSTHESIS, SCROTAL OR INGUINAL APPROACH |
| 54522 | ORCHIECTOMY PARTIAL | ORCHIECTOMY, PARTIAL |
| 54530 | REMOVAL OF TESTIS | ORCHIECTOMY, RADICAL, FOR TUMOR; INGUINAL APPROACH |
| 54535 | EXTENSIVE TESTIS SURGERY | ORCHIECTOMY, RADICAL, FOR TUMOR; WITH ABDOMINAL EXPLORATION |
| 54550 | EXPLORATION FOR TESTIS | EXPLORATION FOR UNDESCENDED TESTIS (INGUINAL OR SCROTAL AREA) |
| 54560 | EXPLORATION FOR TESTIS | EXPLORATION FOR UNDESCENDED TESTIS WITH ABDOMINAL EXPLORATION |
| 54600 | REDUCE TESTIS TORSION | REDUCTION OF TORSION OF TESTIS, SURGICAL, WITH OR WITHOUT FIXATION OF CONTRALATERAL TESTIS |
| 54640 | ORCHIOPEXY INGUN/SCROT APPR | ORCHIOPEXY, INGUINAL OR SCROTAL APPROACH |
| 54650 | ORCHIOPEXY (FOWLER-STEPHENS) | ORCHIOPEXY, ABDOMINAL APPROACH, FOR INTRA-ABDOMINAL TESTIS (EG, FOWLER-STEPHENS) |
| 54660 | REVISION OF TESTIS | INSERTION OF TESTICULAR PROSTHESIS (SEPARATE PROCEDURE) |
| 54670 | REPAIR TESTIS INJURY | SUTURE OR REPAIR OF TESTICULAR INJURY |
| 54680 | RELOCATION OF TESTIS(ES) | TRANSPLANTATION OF TESTIS(ES) TO THIGH (BECAUSE OF SCROTAL DESTRUCTION) |
| 54690 | LAPAROSCOPY ORCHIECTOMY | LAPAROSCOPY, SURGICAL; ORCHIECTOMY |
| 54692 | LAPAROSCOPY ORCHIOPEXY | LAPAROSCOPY, SURGICAL; ORCHIOPEXY FOR INTRA-ABDOMINAL TESTIS |
| 54830 | REMOVE EPIDIDYMIS LESION | EXCISION OF LOCAL LESION OF EPIDIDYMIS |
| 54840 | REMOVE EPIDIDYMIS LESION | EXCISION OF SPERMATOCELE, WITH OR WITHOUT EPIDIDYMECTOMY |
| 54860 | REMOVAL OF EPIDIDYMIS | EPIDIDYMECTOMY; UNILATERAL |
| 54865 | EXPLORE EPIDIDYMIS | EXPLORATION OF EPIDIDYMIS, WITH OR WITHOUT BIOPSY |
| 54900 | FUSION OF SPERMATIC DUCTS | EPIDIDYMOVASOSTOMY, ANASTOMOSIS OF EPIDIDYMIS TO VAS DEFERENS; UNILATERAL |
| 54901 | FUSION OF SPERMATIC DUCTS | EPIDIDYMOVASOSTOMY, ANASTOMOSIS OF EPIDIDYMIS TO VAS DEFERENS; BILATERAL |
| 55040 | REMOVAL OF HYDROCELE | EXCISION OF HYDROCELE; UNILATERAL |
| 55041 | REMOVAL OF HYDROCELES | EXCISION OF HYDROCELE; BILATERAL |
| 55060 | REPAIR OF HYDROCELE | REPAIR OF TUNICA VAGINALIS HYDROCELE (BOTTLE TYPE) |
| 55110 | EXPLORE SCROTUM | SCROTAL EXPLORATION |
| 55120 | REMOVAL OF SCROTUM LESION | REMOVAL OF FOREIGN BODY IN SCROTUM |
| 55150 | REMOVAL OF SCROTUM | RESECTION OF SCROTUM |
| 55175 | REVISION OF SCROTUM | SCROTOPLASTY; SIMPLE |
| 55180 | REVISION OF SCROTUM | SCROTOPLASTY; COMPLICATED |
| 55200 | INCISION OF SPERM DUCT | VASOTOMY, CANNULIZATION WITH OR WITHOUT INCISION OF VAS, UNILATERAL OR BILATERAL (SEPARATE PROCEDURE) |
| 55250 | REMOVAL OF SPERM DUCT(S) | VASECTOMY, UNILATERAL OR BILATERAL (SEPARATE PROCEDURE), INCLUDING POSTOPERATIVE SEMEN EXAMINATION(S) |
| 55400 | REPAIR OF SPERM DUCT | VASOVASOSTOMY, VASOVASORRHAPHY |
| 55500 | REMOVAL OF HYDROCELE | EXCISION OF HYDROCELE OF SPERMATIC CORD, UNILATERAL (SEPARATE PROCEDURE) |
| 55520 | REMOVAL OF SPERM CORD LESION | EXCISION OF LESION OF SPERMATIC CORD (SEPARATE PROCEDURE) |
| 55530 | REVISE SPERMATIC CORD VEINS | EXCISION OF VARICOCELE OR LIGATION OF SPERMATIC VEINS FOR VARICOCELE; (SEPARATE PROCEDURE) |
| 55535 | REVISE SPERMATIC CORD VEINS | EXCISION OF VARICOCELE OR LIGATION OF SPERMATIC VEINS FOR VARICOCELE; ABDOMINAL APPROACH |
| 55540 | REVISE HERNIA & SPERM VEINS | EXCISION OF VARICOCELE OR LIGATION OF SPERMATIC VEINS FOR VARICOCELE; WITH HERNIA REPAIR |
| 55550 | LAPARO LIGATE SPERMATIC VEIN | LAPAROSCOPY, SURGICAL, WITH LIGATION OF SPERMATIC VEINS FOR VARICOCELE |
| 55600 | VESICULOTOMY | VESICULOTOMY; |
| 55605 | VESICULOTOMY COMPLICATED | VESICULOTOMY; COMPLICATED |
| 55650 | REMOVE SPERM DUCT POUCH | VESICULECTOMY, ANY APPROACH |
| 55680 | REMOVE SPERM POUCH LESION | EXCISION OF MULLERIAN DUCT CYST |
| 55720 | DRAINAGE OF PROSTATE ABSCESS | PROSTATOTOMY, EXTERNAL DRAINAGE OF PROSTATIC ABSCESS, ANY APPROACH; SIMPLE |
| 55725 | DRAINAGE OF PROSTATE ABSCESS | PROSTATOTOMY, EXTERNAL DRAINAGE OF PROSTATIC ABSCESS, ANY APPROACH; COMPLICATED |
| 55801 | REMOVAL OF PROSTATE | PROSTATECTOMY, PERINEAL, SUBTOTAL (INCLUDING CONTROL OF POSTOPERATIVE BLEEDING, VASECTOMY, MEATOTOMY, URETHRAL CALIBRATION AND/OR DILATION, AND INTERNAL URETHROTOMY) |
| 55810 | EXTENSIVE PROSTATE SURGERY | PROSTATECTOMY, PERINEAL RADICAL; |
| 55815 | EXTENSIVE PROSTATE SURGERY | PROSTATECTOMY, PERINEAL RADICAL; WITH BILATERAL PELVIC LYMPHADENECTOMY, INCLUDING EXTERNAL ILIAC, HYPOGASTRIC AND OBTURATOR NODES |
| 55821 | REMOVAL OF PROSTATE | PROSTATECTOMY (INCLUDING CONTROL OF POSTOPERATIVE BLEEDING, VASECTOMY, MEATOTOMY, URETHRAL CALIBRATION AND/OR DILATION, AND INTERNAL URETHROTOMY); SUPRAPUBIC, SUBTOTAL, 1 OR 2 STAGES |
| 55831 | REMOVAL OF PROSTATE | PROSTATECTOMY (INCLUDING CONTROL OF POSTOPERATIVE BLEEDING, VASECTOMY, MEATOTOMY, URETHRAL CALIBRATION AND/OR DILATION, AND INTERNAL URETHROTOMY); RETROPUBIC, SUBTOTAL |
| 55840 | EXTENSIVE PROSTATE SURGERY | PROSTATECTOMY, RETROPUBIC RADICAL, WITH OR WITHOUT NERVE SPARING; |
| 55842 | EXTENSIVE PROSTATE SURGERY | PROSTATECTOMY, RETROPUBIC RADICAL, WITH OR WITHOUT NERVE SPARING; WITH LYMPH NODE BIOPSY(S) (LIMITED PELVIC LYMPHADENECTOMY) |
| 55845 | EXTENSIVE PROSTATE SURGERY | PROSTATECTOMY, RETROPUBIC RADICAL, WITH OR WITHOUT NERVE SPARING; WITH BILATERAL PELVIC LYMPHADENECTOMY, INCLUDING EXTERNAL ILIAC, HYPOGASTRIC, AND OBTURATOR NODES |
| 55860 | SURGICAL EXPOSURE PROSTATE | EXPOSURE OF PROSTATE, ANY APPROACH, FOR INSERTION OF RADIOACTIVE SUBSTANCE; |
| 55862 | EXTENSIVE PROSTATE SURGERY | EXPOSURE OF PROSTATE, ANY APPROACH, FOR INSERTION OF RADIOACTIVE SUBSTANCE; WITH LYMPH NODE BIOPSY(S) (LIMITED PELVIC LYMPHADENECTOMY) |
| 55865 | EXTENSIVE PROSTATE SURGERY | EXPOSURE OF PROSTATE, ANY APPROACH, FOR INSERTION OF RADIOACTIVE SUBSTANCE; WITH BILATERAL PELVIC LYMPHADENECTOMY, INCLUDING EXTERNAL ILIAC, HYPOGASTRIC AND OBTURATOR NODES |
| 55866 | LAPS SURG PRST8ECT RPBIC RAD | LAPAROSCOPY, SURGICAL PROSTATECTOMY, RETROPUBIC RADICAL, INCLUDING NERVE SPARING, INCLUDES ROBOTIC ASSISTANCE, WHEN PERFORMED |
| 55873 | CRYOABLATE PROSTATE | CRYOSURGICAL ABLATION OF THE PROSTATE (INCLUDES ULTRASONIC GUIDANCE AND MONITORING) |
| 55875 | TRANSPERI NEEDLE PLACE PROS | TRANSPERINEAL PLACEMENT OF NEEDLES OR CATHETERS INTO PROSTATE FOR INTERSTITIAL RADIOELEMENT APPLICATION, WITH OR WITHOUT CYSTOSCOPY |
| 57240 | ANTERIOR COLPORRHAPHY | ANTERIOR COLPORRHAPHY, REPAIR OF CYSTOCELE WITH OR WITHOUT REPAIR OF URETHROCELE, INCLUDING CYSTOURETHROSCOPY, WHEN PERFORMED |
| 57250 | REPAIR RECTUM & VAGINA | POSTERIOR COLPORRHAPHY, REPAIR OF RECTOCELE WITH OR WITHOUT PERINEORRHAPHY |
| 57265 | CMBN AP COLPRHY W/NTRCL RPR | COMBINED ANTEROPOSTERIOR COLPORRHAPHY, INCLUDING CYSTOURETHROSCOPY, WHEN PERFORMED; WITH ENTEROCELE REPAIR |
| 57284 | REPAIR PARAVAG DEFECT OPEN | PARAVAGINAL DEFECT REPAIR (INCLUDING REPAIR OF CYSTOCELE, IF PERFORMED); OPEN ABDOMINAL APPROACH |
| 57285 | REPAIR PARAVAG DEFECT VAG | PARAVAGINAL DEFECT REPAIR (INCLUDING REPAIR OF CYSTOCELE, IF PERFORMED); VAGINAL APPROACH |
| 57287 | REVISE/REMOVE SLING REPAIR | REMOVAL OR REVISION OF SLING FOR STRESS INCONTINENCE (EG, FASCIA OR SYNTHETIC) |
| 57295 | REVISE VAG GRAFT VIA VAGINA | REVISION (INCLUDING REMOVAL) OF PROSTHETIC VAGINAL GRAFT; VAGINAL APPROACH |
