# Supplementary material for: A pilot intervention to improve the management of urinary tract infections in outpatient settings
Source: Antimicrob Steward Healthc Epidemiol. 2025 Dec 18;5(1):e338. doi: 10.1017/ash.2025.10228 (PMC12722549; doi:10.1017/ash.2025.10228)
Supplement: Madaras-Kelly et al. supplementary material 4 — Madaras-Kelly et al. supplementary material [file S2732494X25102283sup004.docx]

**Supplement 3. Chart Validation Results**

| **Reviewer** | **Clinician** | | | | | | | |
| --- | --- | --- | --- | --- | --- | --- | --- | --- |
|  |  | **Cystitis/UTI** | **Other GU Infection** | **Non-infectious GU Dx** | **Symptoms of GU condition** | **ASB** | **Other** | **Total** |
|  | **Cystitis/UTI** | **68** | 0 | 4 | 3 | 0 | 2 | **77** |
|  | **Other GU Infection** | 7 | **19** | 1 | 2 | 0 | 0 | 29 |
|  | **Non-infectious GU Dx** | 24 | 10 | **177** | 16 | 0 | 11 | 238 |
|  | **Symptoms of GU condition** | 6 | 2 | 8 | **51** | 0 | 8 | 75 |
|  | **ASB** | 20 | 0 | 3 | 1 | **0** | 4 | 28 |
|  | **Other** | 7 | 0 | 8 | 3 | 0 | **46** | 64 |
|  | **Total** | 132 | 31 | 201 | 76 | 0 | **71** | **511** |

**Legend:** GU: Genitourinary, ASB asymptomatic bacteriuria, UTI: urinary tract infection.
