# Supplementary material for: A pilot intervention to improve the management of urinary tract infections in outpatient settings
Source: Antimicrob Steward Healthc Epidemiol. 2025 Dec 18;5(1):e338. doi: 10.1017/ash.2025.10228 (PMC12722549; doi:10.1017/ash.2025.10228)
Supplement: Madaras-Kelly et al. supplementary material 5 — Madaras-Kelly et al. supplementary material [file S2732494X25102283sup005.docx]

**Supplement 4. Diagnostic Shifting Results**

| **Individual Genitourinary Diagnosis Shifts** | | | | | | |
| --- | --- | --- | --- | --- | --- | --- |
|  | **Intervention Sites** | | | **Control Sites** | | |
| **Diagnostic Group*** | **Pre-Implementation, *N*(%)** | **Post-Implementation,**  ***N*(%)** |  | **Pre-Implementation, *N*(%)** | **Post-Implementation, *N*(%)** | **aRR**  **(+95% CI)** |
| Metric A | 28,218 (100) | 29,221(100) | **RR**  **(+95% CI)** | 45,989 (100) | 47,077 (100) | **RR**  **(+95% CI)** |
| Metrics C&D (uUTI) | 1,190 (4.2) | 1,203 (4.1) | **Reference** | 2,261 (4.9) | 2,396 (5.1) | **Reference** |
| Other Tier 1 | 831 (2.9) | 814 (2.8) | 0.99 (0.94-1.05) | 1,178 (2.6) | 1,174 (2.5) | 1.01 (0.95-1.06) |
| Metric B | 21,406(75.6) | 22,068 (75.5) | 1.00 (0.99-1.00) | 35,133 (76.4) | 36,060 (76.6) | 1.00 (0.99-1) |
| Metric B non-R codes | 18,368 (65.1) | 19,060 (65.2) | 1.00(0.99-1.01) | 30,794 (67.0) | 31,442 (66.8) | 1.00 (0.99-1.001) |
| Metric B, R codes | 3,038 (10.8) | 3,008 (10.3) | 1.01 (0.97-1.04) | 4,339 (9.4) | 4,618 (9.8) | 1.02 (0.99-1.06) |
